# Overall Survival Benefits of Cancer Drugs Approved in China From 2005 to 2020

**DOI:** 10.1001/jamanetworkopen.2022.25973

**Published:** 2022-08-10

**Authors:** Yichen Zhang, Huseyin Naci, Anita K. Wagner, Ziyue Xu, Yu Yang, Jun Zhu, Jiafu Ji, Luwen Shi, Xiaodong Guan

**Affiliations:** 1 Department of Pharmacy Administration and Clinical Pharmacy, School of Pharmaceutical Sciences, Peking University, Beijing, China; 2 LSE Health, Department of Health Policy, London School of Economics and Political Science, London, United Kingdom; 3 Department of Population Medicine, Harvard Medical School and Harvard Pilgrim Health Care Institute, Boston, Massachusetts; 4 Beijing Cancer Hospital, Beijing, China; 5 International Research Centre for Medicinal Administration, Peking University, Beijing, China

## Abstract

**Importance:**

Of approximately 9 million patients with cancer in China in 2020, more than half were diagnosed with late-stage cancers. Recent regulatory reforms in China have focused on improving the availability of new cancer drugs. However, evidence on the clinical benefits of new cancer therapies authorized in China is not available.

**Objective:**

To characterize the clinical benefits of cancer drugs approved in China, as defined by the availability and magnitude of statistically significant overall survival (OS) results.

**Design, Setting, and Participants:**

This mixed-methods study comprising a systematic review and cross-sectional analysis identified antineoplastic agents approved in China between January 1, 2005, and December 31, 2020, using publicly available data and regulatory review documents issued by the National Medical Products Administration. The literature published up to June 30, 2021, was reviewed to collect results on end points used in pivotal trials supporting cancer drug approvals.

**Main Outcomes and Measures:**

The primary outcome measure was a documented statistically significant positive OS difference between a new cancer therapy and a comparator treatment. Secondary outcome measures were the magnitude of OS benefit and other primary efficacy measures in pivotal trials.

**Results:**

Between 2005 and 2020, 78 cancer drugs corresponding to 141 indications were authorized in China, including 20 drugs (25.6%) (for 30 indications) approved in China only. Of all indications, 26 (18.4%) were evaluated in single-arm or dose-optimization trials, most of which were authorized after 2017. By June 30, 2021, 34 drug indications (24.1%) had a documented lack of OS gain. For 68 indications (48.2%) that had documented evidence of OS benefit, the median magnitude of OS improvement was 4.1 (range, 1.0-35.0) months. After a median follow-up of 1.9 (range, 1.0-11.1) years from approval, OS data for 13 indications (9.2%) were either not reported or were still not mature. Fewer than one-third of cancer drug indications approved in China only had documented evidence of OS benefits (9 of 30 [30.0%]), whereas more than one-half of the cancer drug indications also available in the US or Europe had OS benefits (59 of 111 [53.1%]).

**Conclusions and Relevance:**

In this study, almost half of cancer drug indications approved in China had demonstrated OS gain. With the increase of cancer drug approvals based on single-arm trials or immature survival data in recent years, these findings highlight the need to routinely monitor the clinical benefits of new cancer therapies in China.

## Key Points

**Question:**

What is the overall survival benefit of cancer drugs approved in China between 2005 and 2020?

**Findings:**

In this mixed-methods study comprising a systematic review and cross-sectional analysis of 78 cancer drugs for 141 indications, 68 new cancer drug indications approved by Chinese authorities between 2005 and 2020 had documented evidence of overall survival benefit, and 34 did not prolong life.

**Meaning:**

Given regulatory reforms in recent years, these findings highlight the need to routinely monitor the clinical benefits of new cancer therapies in China.

## Teaser

This systematic review characterizes cancer drugs approved in China from 2005 to 2020 and investigates their clinical benefits in terms of availability and magnitude of documented overall survival benefits.

## Introduction

The primary goal of cancer drug therapy is to prolong life or to improve quality of life.^1,2^ Overall survival (OS) is the most reliable clinical trial end point to inform regulatory approvals of new cancer drugs.^3,4^ However, most cancer drugs approved in the US and Europe lack evidence of OS benefit. For instance, more than two-thirds of cancer drug approvals granted by the US Food and Drug Administration (FDA) between 2008 and 2012 were based on surrogate end points, and only 14% of drugs initially approved on the basis of surrogate end points had demonstrated OS benefits after a median follow-up of 4.4 years.^5^ Further, drugs met the threshold for clinically meaningful benefit in less than half of trials supporting solid tumor indication approvals by the FDA between 2006 and 2016.^6^ Similarly, among cancer drugs approved by the European Medicines Agency (EMA) between 2009 and 2013, only 51% showed significant improvement of survival or quality of life at a minimum of 3.3 years follow-up, and survival gains over existing therapy were marginal.^7^

**Figure 1.**
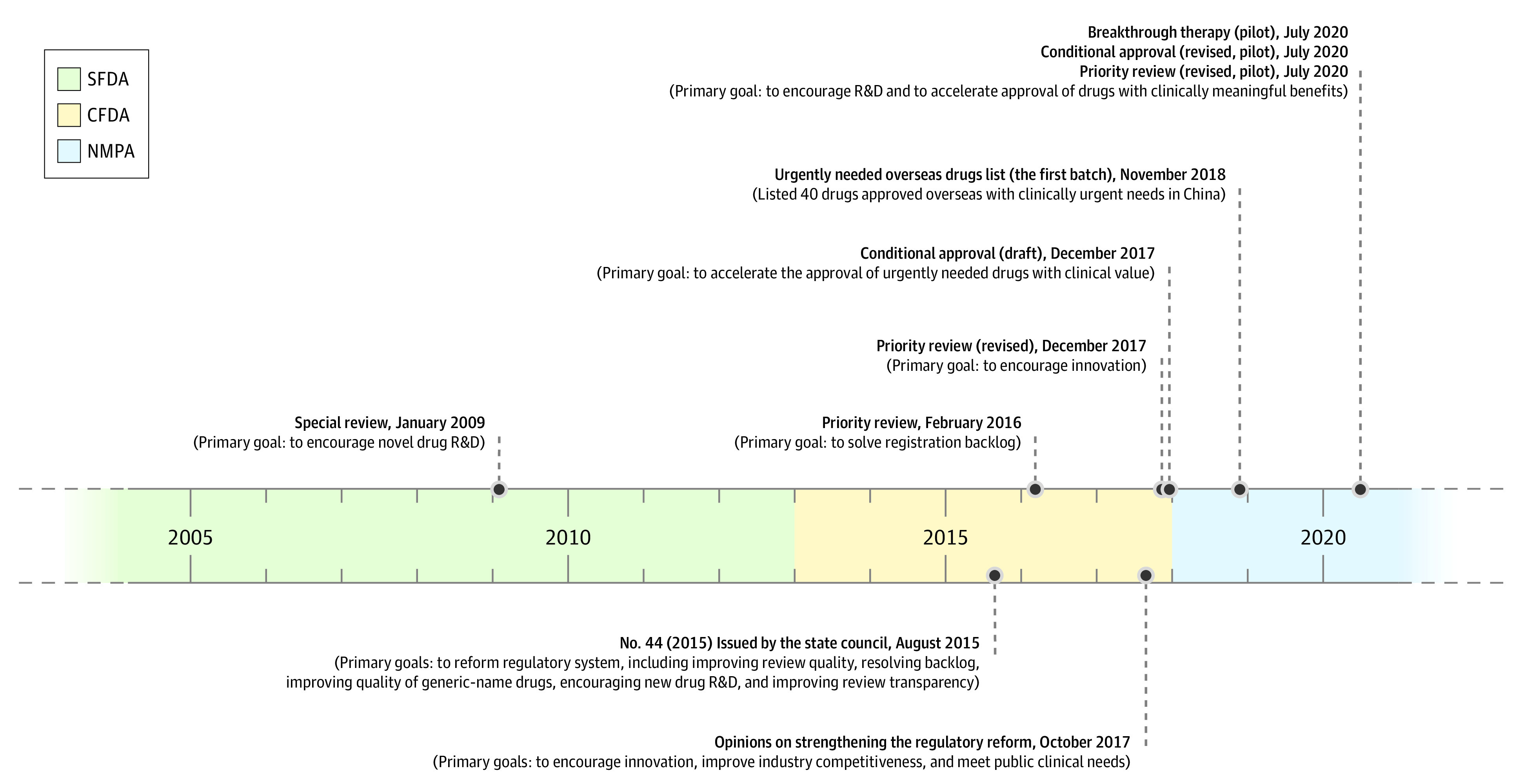
Timeline of Landmark Legislation and Regulations Relating to China’s Drug Administration Landmark policies above the timeline were issued by the National Medical Products Administration (NMPA) (also called State Food and Drug Administration [SFDA] and previously the China Food and Drug Administration [CFDA]). Policies below the timeline were issued by the State Council. See eTable 1 in the Supplement for more details of the policies. R&D indicates research and development.

Cancer is the leading cause of death in China.^8^ However, the availability of effective novel cancer drugs has remained limited owing to widely documented regulatory delays during the past decades.^9,10^ In recent years, there has been a concerted effort to improve the availability of new cancer medicines in China (Figure 1).^11^ China has overhauled its drug regulatory agency, the China Food and Drug Administration (CFDA), now called the National Medical Products Administration (NMPA).^12^ Under the CFDA and NMPA, the government has expanded regulatory capacity^13^ and created a series of programs to expedite the development, review, and approval of new cancer drugs.^14,15^ These programs closely parallel those established by the FDA and EMA, which allow drug approval based on less complete data than traditionally required. Similar to US and European Union (EU) regulators, Chinese regulators have introduced flexibility for using surrogate end points to support drug approvals under certain conditions, such as those for serious or life-threatening diseases, especially if new drugs appear superior to existing treatments.^2,16,17,18^ In 2017, several additional reforms were implemented to incentivize China’s domestic research and development capacity.^19^

Recent regulatory changes in China have effectively encouraged investment in drug research and development, and oncology trial activity has increased 10-fold.^20,21^ The number of new cancer drug approvals, especially those developed and initially approved in China, has increased substantially during the past 5 years.^22^ However, to our knowledge, no recent studies have systematically examined the characteristics of cancer drugs authorized in China during the past decades, and the clinical benefits of cancer therapies approved by the NMPA remain unknown. In this study, we characterized cancer drugs approved in China from 2005 to 2020 and investigated their clinical benefits in terms of availability and magnitude of documented OS benefits.

## Methods

Using publicly available data, we curated a novel database of cancer drug approvals between January 1, 2005, and December 31, 2020, to summarize the characteristics and results of clinical trials supporting regulatory approvals in China. We start the database in 2005 because the Center for Drug Evaluation issued the first guideline on clinical trial results reporting at that time.^11^ We distinguished between cancer drugs approved in China only and those also approved by the FDA or the EMA. We determined whether drugs had evidence of OS benefit for the approved indications. For drugs targeting solid tumors also authorized by the FDA or the EMA, we further evaluated whether they had clinically meaningful benefits using the validated European Society for Medical Oncology–Magnitude of Clinical Benefit Scale (ESMO-MCBS) scorecard. This mixed-methods study comprising a systematic review and cross-sectional analysis adheres to the Preferred Reporting Items for Systematic Reviews and Meta-Analyses (PRISMA) reporting guidelines and relevant portions of the Strengthening the Reporting of Observational Studies in Epidemiology (STROBE) guidelines.

### Data Sources and Sample Selection

Four information sources were used to construct the database, matching cancer drug indication approvals with supporting evidence. The National Drug Code Data File contains all medical products available on the Chinese market with information including generic name, formulation, strength, and manufacturer name. Drug labels were obtained from the publicly available data provided by the Center for Drug Evaluation,^23,24^ manufacturer websites, and the Beijing Cancer Hospital.^25^ Regulatory review documents summarize the evidence supporting indication approvals. Not all product labels and regulatory review documents were publicly available through June 30, 2021.^23^ We therefore cross-checked our sample of cancer drug indications using a commercial data set that contains information on authorized and investigational drugs.^26^ This data set was also used in previous studies.^22,27^

Using the National Drug Code Data File issued by the NMPA,^28^ we identified by generic name all cancer drugs approved in China between January 1, 2005, and December 31, 2020. We included patent-protected small molecular entities as well as biologics. Traditional Chinese medicines, vaccines, and diagnostic products were excluded. If the FDA- or EMA-approved originator version of a specific active ingredient had not been approved in China and a generic version was, we included the first generic product approval. Medicines without a clinical trials section in their labels were also excluded. Cancer drugs were categorized as those approved in China only and those that were also approved in the US^29^ or Europe^30^ by December 31, 2020. All sample cancer drugs were classified into chemotherapy, radiotherapy drug, hormone therapy, targeted therapy, and immunotherapy.

For each cancer drug in our sample, we reviewed its latest label and regulatory review documents (by June 30, 2021) to identify approved indications. We excluded indications for pediatric use only, benign tumors, precancerous lesions, and supportive care (see eFigure 1 in the Supplement).^31^ All sample cancer drug indications were categorized into first-line, later-line, and adjuvant or neoadjuvant therapies.

### Identification of Pivotal Trials

For each indication, we identified the pivotal trials supporting regulatory approval. When the regulatory review document was unavailable, we identified all premarketing trials included in the Clinical Trials section of the latest label through December 31, 2020.^32^ Phase 1 studies were excluded when phase 2 or 3 trials were available. We used ClinicalTrials.gov (US National Library of Medicine database of clinical trials) and ChinaDrugsTrials.org (the official trial registration website of the Center for Drug Evaluation and NMPA) to identify study details.^21,33^ We then searched the study names and clinical trial identifiers in PubMed, China National Knowledge Infrastructure, and ClinicalTrials.gov to retrieve peer-reviewed publications of pivotal trials published by June 30, 2021 (eBox 1 in the Supplement includes more details). We also searched the reference lists of relevant reports for previously unidentified studies if no records were identified from bibliographic databases and clinical trial registries. Two investigators (Z.X. and Y.Y.) independently reviewed the titles and abstracts of all records to identify the primary publications reporting trial results. Another investigator (Y.Z.) resolved disagreements.

Using available regulatory review documents, labels, and identified publications, we extracted OS data at the following points within each trial to check whether a statistically significant OS benefit was demonstrated before regulatory approval at the following dates: (1) every cutoff date for data analysis (eg, database lock date for preplanned interim analysis, updated analysis, and final OS analysis); (2) initial publication date (ie, the online publication date if available); and (3) regulatory approval date. If data reported in the published study conflicted with the regulatory review document at the same time point, we relied on the data reported in the regulatory review document. Information on other primary and secondary end points, including progression-free survival and tumor response rates, was also extracted (eTable 2 in the Supplement). For randomized clinical trials, the comparators were classified into chemotherapy, targeted therapy, immunomodulating therapy, best supportive care, conventional care, add-on (in trials comparing drugs A plus B vs B alone), or placebo.

To evaluate whether a cancer therapy showed a survival benefit, we categorized OS data into 4 categories: (1) documented OS benefit for which the drug had a statistically significant association with OS in at least one of the pivotal trials; (2) documented lack of OS benefit, for which the drug did not prolong survival in any supporting pivotal trials (ie, there was no statistically significant difference between the intervention and control groups); (3) OS benefit unknown, for which OS was either the primary end point or was included among trial end points, but OS data was immature or not reported in any publications by the end of our data collection (June 30, 2021); and (4) OS benefit not evaluable, for which indications were supported only by single-arm or dose-optimization trials that did not have comparators (see eTable 3 in the Supplement for further details).

### Association Between Surrogate End Points and OS

For indications that did not fall into the category of documented OS benefit, we identified surrogate end points, cancer sites, and cancer treatment settings in pivotal trials. For each surrogate end point used in a specific cancer site and cancer treatment setting combination, we extracted the correlation coefficients with OS from a recent umbrella review.^34^ If the combination was not included in the umbrella review, we searched for additional studies evaluating the association between surrogate end points and OS (eBox 2 in the Supplement includes further details on search strategy). The correlation between surrogate end points and OS was categorized as high (*r* ≥ 0.85), medium (*r* = 0.70 to <0.85), and low (*r* ≤ 0.70), in line with previous studies.^34,35,36^

### Statistical Analysis and Sensitivity Analysis

We used descriptive statistics to characterize the number of cancer drugs and indications by approval year, cancer type, and OS benefit category. For each trial, we determined whether OS benefit was shown before approval. We also documented the magnitude of OS benefit or other primary efficacy end points. If a drug was supported by more than 1 trial, the most favorable result was used to calculate the median benefit.

For drugs indicated for solid tumors, the validated ESMO-MCBS scorecard was used to assess their therapeutic value.^37^ Scores A to B in the curative setting or 4 or 5 in the noncurative setting were considered a clinically meaningful benefit.^38^ For therapies with unknown OS data, we calculated the median time elapsed between regulatory approval and the end of our data collection (June 30, 2021).

## Results

### Characteristics of Indications and Supporting Trials

**Figure 2.**
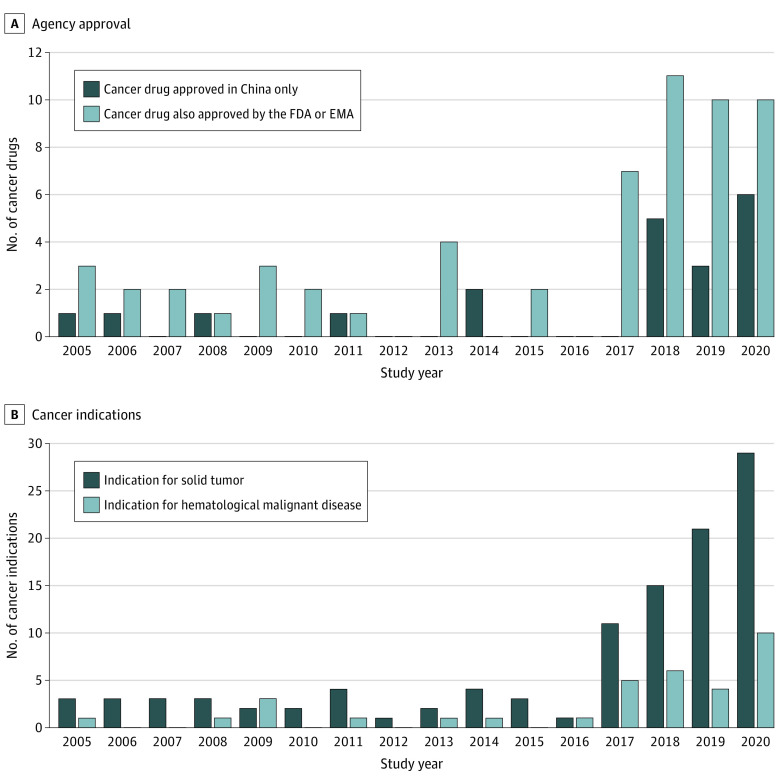
First Marketing Authorization and Supplemental Indication Approval Time of Cancer Drugs Authorized in China Between 2005 and 2020 A, Cancer drugs approved in China only and cancer drugs also approved by the US Food and Drug Administration (FDA) or the European Medicines Agency (EMA) by December 31, 2020. B, Indications for solid tumors or hematological malignant neoplasms.

From 2005 to 2020, 82 new cancer drugs were authorized in China. After exclusions, 78 cancer drugs corresponding to 141 indications were included in our study. Of these, 52 drugs (66.7%) corresponding to 101 indications (71.6%) were approved between 2017 and 2020 (Figure 2). Twenty drugs (25.6%) (Figure 2A) for 30 indications were authorized in China only, and 14 (70.0%) of these were approved after 2017; 15 of 20 (75.0%) were targeted therapies, and 4 of 20 (20.0%) were immunotherapies. In addition to regulatory review documents and labels (see eTable 4 in the Supplement), we also identified 241 pivotal trials in 395 publications from which we extracted data.

Figure 2B and Table 1 show the characteristics of the cancer drug indications. Most indications (107 [75.9%]) were for solid tumors, and 34 (24.1%) were for hematological malignant neoplasms. The most common cancer types were lung cancers (31 [22.0%]), followed by lymphoma (16 [11.3%]), breast cancers (14 [9.9%]), and leukemia (12 [8.5%]). Most indications (115 [81.6%]) were supported by at least 1 randomized clinical trial; 26 (18.4%) were supported by single-arm or dose-optimization studies. Most indications for solid tumors (98 [91.6%]) were supported by randomized clinical trials; half of the hematological malignant indications (17 [50.0%]) were approved based on single-arm trials. Of the 26 indications approved based on noncomparative trials, 20 (76.9%) were authorized after 2017 (eFigure 2 in the Supplement).

**Table 1.** Characteristics of Cancer Drug Indications Approved by China’s National Medical Products Administration, 2005 to 2020

Characteristic	Indications, No. (%)		
	All (n = 141)	Pivotal trial design^a^	
		Supported by randomized clinical trial (n = 115)	Supported by single-arm or dose-optimization trial only (n = 26)
Tumor type			
Solid	107 (75.9)	98 (91.6)	9 (8.4)
Hematological malignant neoplasm	34 (24.1)	17 (50.0)	17 (50.0)
Market authorization			
Approved in China only	30 (21.3)	16 (53.3)	14 (46.7)
Also approved by FDA or EMA^b^	111 (78.7)	99 (89.2)	12 (10.8)
Cancer drug type			
Chemotherapy	15 (10.6)	13 (86.7)	2 (13.3)
Radiotherapy drug	2 (1.4)	1 (50.0)	1 (50.0)
Hormone therapy	8 (5.7)	8 (100)	0
Targeted therapy	96 (68.1)	79 (82.3)	17 (17.7)
Immunotherapy	20 (14.2)	14 (70.0)	6 (30.0)
Cancer site			
Lung	31 (22.0)	27 (87.1)	4 (12.9)
Lymphoma	16 (11.3)	4 (25.0)	12 (75.0)
Breast	14 (9.9)	14 (100)	0
Leukemia	12 (8.5)	8 (66.7)	4 (33.3)
Prostate	8 (5.7)	8 (100)	0
Liver	7 (5.0)	5 (71.4)	2 (28.6)
Colon and rectum	7 (5.0)	7 (100)	0
Melanoma	7 (5.0)	6 (85.7)	1 (14.3)
Multiple myeloma	6 (4.3)	5 (83.3)	1 (16.7)
Kidney	5 (3.5)	5 (100)	0
Ovary	5 (3.5)	4 (80.0)	1 (20.0)
Neuroendocrine tumor	4 (2.8)	4 (100)	0
Stomach	3 (2.1)	3 (100)	0
Brain	3 (2.1)	3 (100)	0
Head and neck	3 (2.1)	3 (100)	0
Esophagus	2 (1.4)	2 (100)	0
Thyroid	2 (1.4)	2 (100)	0
Gastrointestinal stromal tumor	2 (1.4)	2 (100)	0
Urothelial	1 (0.7)	0	1 (100)
Nasopharyngeal	1 (0.7)	1 (100)	0
Soft tissue sarcoma	1 (0.7)	1 (100)	0
Mesothelioma	1 (0.7)	1 (100)	0

Abbreviations: EMA, European Medicine Agency; FDA, US Food and Drug Administration.

^a^ Calculated as row percentages.

^b^ By December 31, 2020.

Among 30 indications for 20 cancer drugs approved in China only, 14 (46.7%) were authorized based on single-arm trials. Ninety-nine of 111 indications (89.2%) for cancer drugs also approved by the FDA or EMA were supported by randomized clinical trials s (Table 1 and Table 2). Indications for cancer drugs approved in China only and those also approved by the FDA or EMA had the same rank of the most common 3 cancer types (lung, lymphoma, and breast). Approximately one-quarter of drug indications approved only in China (8 [26.7%]) were for first-line therapy, and the remaining 22 (73.3%) were for later lines of therapy (eTable 5 in the Supplement).

**Table 2.** Classification of OS Benefit Evidence of Cancer Drug Indications Approved by China’s National Medical Products Administration, 2005 to 2020

OS benefit evidence	Indication, No. (%)^a^		
	All (n = 141)	Cancer drugs approved in China only (n = 30)^b^	Cancer drugs also approved by FDA or EMA (n = 111)^b^
Documented OS benefit	68 (48.2)	9 (30.0)	59 (53.1)
Documented lack of OS benefit	34 (24.1)	3 (10.0)	31 (27.9)
OS benefit unknown	13 (9.2)	4 (13.3)	9 (8.1)
OS benefit not evaluable	26 (18.4)	14 (46.7)	12 (10.8)

Abbreviations: EMA, European Medicine Agency; FDA, US Food and Drug Administration; OS, overall survival.

^a^ Percentages have been rounded and may not total 100.

^b^ By December 31, 2020.

### Classification of OS Benefit

Overall, drugs for 68 cancer indications (48.2%) demonstrated a statistically significant prolongation of survival. Nearly all drug indications with OS benefit (n = 65) had shown the benefit before market authorization in China (OS data matured after NMPA approval for the following 3 indications: crizotinib in ALK mutation–positive advanced or metastatic non–small cell lung cancer, alectinib in ALK mutation–positive advanced or metastatic non–small cell lung cancer, and bortezomib for mantle-cell lymphoma). For 34 indications (24.1%), a drug’s lack of a statistically significant OS benefit was documented before approval (except for osimertinib for second-line therapy in epidermal growth factor receptor T790M–positive advanced non–small cell lung cancer, for which OS data matured after approval). Compared with drug indications approved only in China, a higher proportion of cancer drug indications also authorized in the US or the EU had evidence of survival benefit: 59 of 111 (53.1%) vs 9 of 30 (30.0%) (Table 2). Over time, there was no apparent change in the proportion of authorized therapies with evidence of OS benefit (eFigure 3 in the Supplement). Fewer than one-third of cancer drug indications approved in China only had documented evidence of OS benefits (9 of 30 [30.0%]), whereas more than one-half of the cancer drug indications also available in the US or Europe had OS benefits (59 of 111 [53.1%]).

By the end of our study period (June 30, 2021), with a median follow-up of 1.9 (range, 1.0-11.1) years after approval, OS outcomes were unknown for 13 indications (9.2%), including 7 for which OS data were immature in the latest reported result and 6 for which OS data were not mentioned in any publication, regulatory document, or label. In addition, 25 indications were approved based on single-arm studies and 1 was approved based on a dose-optimization study (Table 2).

Among 107 indications for solid tumor therapies that could be scored by ESMO-MCBS, 41 did not have publicly available scores, including 19 indications authorized by neither the FDA nor the EMA. Of 66 therapies scored by ESMO-MCBS, 38 (57.6%) were judged as having clinically meaningful benefit. Thirteen of 42 indications with documented OS benefit (30.9%) did not have clinically meaningful benefit, and 8 of 18 indications with documented lack of OS benefit (44.4%) were classified as clinically meaningful by ESMO-MCBS (eTable 6 in the Supplement).

### Magnitude of OS Benefit and Other Primary Efficacy Measures

Of the 68 indications with documented statistically significant OS benefit, 45 (66.2%) had placebo or add-on comparators, 13 (19.1%) were chemotherapy, 8 (11.8%) were targeted therapy, and 2 (2.9%) had other comparators in their pivotal trials. Compared with the control arm, the median improvement in OS was 4.1 months, ranging from 1.0 to 35.0 months (eTable 7 in the Supplement).

Pivotal trials of the 34 indications with documented lack of OS benefit measured progression-free survival (25 [73.5%]) or other surrogate outcomes as their primary end points, and 16 (47.1%) had placebo or add-on comparators. Effect sizes varied across studies, ranging from no progression-free survival difference between the intervention and control arms to 36.1 (range, 0-36.1) months progression-free survival gain (eTable 8 in the Supplement).

Of the 13 indications for which OS data were immature or not reported, 10 (76.9%) were approved between 2017 and 2020. Of 26 indications supported only by noncomparative trials, 17 (65.4%) Table 1) were approved for the treatment of hematological malignant neoplasms. The most common end point (23 [88.5%]) was the objective response rate (ORR), usually defined as the proportion of patients achieving complete response or partial response. Among the 26 indications, the median ORR was 69.1%, ranging from 8.2% to 87.1%, with the complete response rate ranging from 0 to 68.6% and the partial response rate from 1.8% to 65.5% (eTable 9 and 10 in the Supplement).

### Correlation Between Surrogate End Points and OS

Overall, among 73 indications without documented OS benefit, we could not find any published study evaluating the association between surrogate end points and OS in 35 cancer indications (47.9%) (Table 3; see eTable 11 in the Supplement for further details). For surrogate end points supporting 38 indications (52.1%), published evaluations reported a high correlation with OS for 10 indications (26.3%), moderate correlation for 7 (18.4%), and low correlation for 21 (55.3%) (Table 3).

**Table 3.** Correlations Between Surrogate End Points and OS Among Cancer Indications Without Documented OS Benefit

Correlation between surrogate end points and OS^a^	Indication, No. (%)			
	Without documented OS benefit (n = 73)	Documented lack of OS benefit (n = 34)	OS benefit unknown (n = 13)	OS benefit not evaluable (n = 26)
Published surrogate correlation studies^b^	38 (52.1)	23 (67.6)	7 (53.8)	8 (30.8)
High (*r*≥0.85)	10 (26.3)	6 (26.1)	4 (57.1)	0
Medium (*r* = 0.7 to <0.85)	7 (18.4)	5 (21.7)	1 (14.3)	1 (12.5)
Low (*r*≤0.7)	21 (55.3)	12 (52.2)	2 (28.6)	7 (87.5)
No published surrogate correlation studies^b^	35 (47.9)	11 (32.3)	6 (46.1)	18 (69.2)

Abbreviation: OS, overall survival.

^a^ Correlation information based on published literature (see eTable 11 in the Supplement for further detail).

^b^ For specific cancer type and line of therapy.

## Discussion

### Principal Findings

To our knowledge, this is the first review to systematically evaluate the characteristics and clinical benefits of cancer drugs authorized in China. Our results show that between 2005 and 2020, 78 new antineoplastic agents were approved in China for 141 indications. Most approvals occurred after 2017, and approximately one-fifth of approvals were supported by single-arm trials only. Compared with control treatments, new therapies for 68 indications statistically significantly prolonged patient survival, with a median survival improvement of 4.1 (range, 1.0-35.0) months. For the other 73 indications without documented OS gain at the time of approval, only 10 were approved based on the surrogate end points with a high correlation with OS, and the magnitude of differences in surrogate end points between intervention and comparator groups varied.

### Comparison With Other Studies

We characterized cancer drug approvals during a 16-year period when regulatory reforms were implemented to increase the number of new cancer drugs in China. Previous studies highlighted China’s overreliance on imported medicines^19^ and found an association between the regulatory reforms and increases in research and development investment and clinical trial activity.^21,27,39^ As suggested by our findings, the number of cancer drug approvals has increased sharply since 2017, reaching levels similar to those in the US and the EU.^40^ Between 2017 and 2020, 52 new cancer drugs were authorized in China, including 14 approved only in China. During that time, 57 new cancer drugs were approved in the US and 45 in the EU.^41,42^

Historically, regulators have considered OS as the criterion standard for establishing cancer drug efficacy.^4,43,44^ Longer survival is also the preferred end point by most patients with metastatic cancers.^45^ During our study period, approximately half of new cancer drug indications had documented statistically significant OS benefits. We also observed growing regulatory reliance on surrogate end points for approving cancer drugs in China, similar to trends observed in the US and Europe.^46,47,48^ Between 2005 and 2020, approximately one-third of cancer drug indications were approved on the basis of surrogate end points alone. Moreover, our investigation also reveals an upward trend since 2017 of cancer indication approvals based on single-arm trials with ORR as the primary end point. The median response rate for cancer indications authorized in China based on single-arm trials (median ORR, 69.1% across 29 indications) seems similar to those for drugs approved in the US (median response rate, 41.0% across 85 indications)^49^ and in Europe (median ORR, 55.6% across 22 indications).^50^ Previous literature has shown drug benefits on surrogate end points often do not reliably estimate the desired clinical outcomes for patients: improved survival or quality of life.^35,36,51,52^ Of concern, our results indicate that more than one-third of indications approved in China without documented OS benefit were approved based on surrogate end points with reported medium or low correlations with OS. Ten indications were approved based on surrogate end points with high correlations with OS in the given settings, and approximately one-quarter of indications with surrogate measures as primary trial end points did not improve patient survival.

### Implications for Patients, Clinicians, and Policy Makers

Results of the present study have important implications for patients, clinicians, and policy makers. Patients with cancer expect that new drugs prescribed by their physicians will help them live better for longer.^53,54^ Currently, patients lack information about the limited evidence of benefits of approved cancer drugs and may have misconceptions about efficacy and safety, creating false hopes for benefits and underestimating risks.^55,56^ Clinicians’ codes of professional ethics require that they support each patient in making decisions about the risk-benefit balance of a therapy. When prescribing new drugs with uncertain benefits and risks, physicians need to communicate to their patients the uncertainty of clinical benefits and potential toxic effects of new therapies.^57^ To support clinicians in their obligation to convey what is known and what is not known about new cancer drugs and support patient-clinician decision making, regulators should mandate consistent documentation of overall survival benefit information in approved drug labeling^58^ and could add clear visual alerts in labels of limited evidence of benefits of akin to black box warnings in FDA-approved drug labels.^59^

In recent years, Chinese regulators have created several expedited drug approval pathways that resemble those in the US and Europe. Some of the latest reforms in 2020 added oncology-specific expedited approval regulations. Although expedited pathways may shorten the duration of clinical development and regulatory review,^60^ benefits of such pathways should be weighed against the harms of allowing drugs with limited clinical benefits to enter the market. Studies in other countries have highlighted inadequacies of timely completion and reporting of postmarketing trials.^61^ Data limitations at the time of regulatory approval may complicate estimation of cost-effectiveness for reimbursement decisions,^62^ which may lead to potential waste of scarce public insurance resources.^63,64^ Because regulatory approval standards represent not only the current thinking of regulators, but also guide the vision of what a “good drug” should be,^65^ our findings highlight the need for more attention to the clinical meaningfulness—in terms of type and magnitude of benefit^66^—of newly authorized and investigational cancer therapies in China.^67,68^

### Limitations

This study has several limitations. First, Chinese authorities do not disclose every version of the label. It is therefore possible that we missed newly approved cancer drug indications during our study period. However, we used a multipronged approach to collect and validate approval information from different sources. Second, because the NMPA does not routinely disclose the evidence base supporting its decisions, pivotal trial information supporting 54 of 141 indications (38.3%) was obtained from product labels. We compared regulatory review documents and labels and found that key phase 2 and 3 pivotal trials documented in both were the same. Similarly, we excluded 3 cancer drugs for which clinical trial information supporting approvals was not disclosed. Third, publicly available data may represent a selective subset of clinical trial information available for new cancer drugs.^69,70^ Because not all trials have published updated results,^71^ the proportion of indications with unknown OS may be overestimated. Fourth, cancer drugs approved in China only and drugs for hematological malignant disease do not have ESMO-MCBS scores, so that only 66 of 141 indications (46.8%) could be matched with ESMO-MCBS scores, which may lead to underestimating the proportion of new therapies with clinically meaningful benefit.

## Conclusions

Fewer than half of new cancer therapies approved in China between 2005 and 2020 had documented OS benefits. For half of the indications without documented OS benefit, the correlation of surrogate end points with OS was unknown. Only for 14% did the published literature suggest a high correlation between surrogate end points and OS. With the increased use of surrogate end points and single-arm trials supporting regulatory approvals, our findings highlight the need to create awareness of and enhance the evidence of clinical benefits of cancer drugs in China.

## Notes

Supplement. **eTable 1.** Landmark Regulatory Policies Concerning Drug Approvals in China, 2009-2020 **eFigure 1.** Identification of Sample Indications **eBox 1.** Key Words and Research Strategy to Identify Peer-Reviewed Publications of Drug Trials **eTable 2.** Outcome Variables Extracted From Trials **eTable 3.** Classification of Overall Survival (OS) Benefit **eBox 2.** Key Words and Research Strategy to Identify Peer-Reviewed Publications of Correlations Between Surrogate End Points and Overall Survival **eTable 4.** Sources of Information **eFigure 2.** Approval Year of Indications Supported Only by Single-Arm or Dose-Optimization Trials **eFigure 3.** Classification of Overall Survival (OS) Benefit for Cancer Indications Approved in China, by Approval Year **eTable 5.** Line of Therapy of Cancer Indications of Drugs Approved in China Only and Drugs Also Approved in the United States or the European Union **eTable 6.** Classification of Overall Survival and ESMO-MCBC Score of Cancer Drugs **eTable 7.** Magnitude of Documented Overall Survival (OS) Benefit for 68 Indications, by Cancer Type **eTable 8.** Results of Pivotal Trials Supporting 34 Indications With Documented Lack of Statistically Significant Overall Survival (OS) Benefit **eTable 9.** Primary End Point and Time From Enrollment to Approval of 13 Indications With Unknown Overall Survival Data by June 30, 2021 **eTable 10.** Results of Single-Arm or Dose-Optimization Trials Supporting Approvals by China National Medical Products Administration, 2005-2020 **eTable 11.** Published Surrogate Correlation Studies for Cancer Indications Without Documented Overall Survival Benefit
References
