# Supplementary material for: Overall Survival Benefits of Cancer Drugs Approved in China From 2005 to 2020
Source: JAMA Netw Open. 2022 Aug 10;5(8):e2225973. doi: 10.1001/jamanetworkopen.2022.25973 (PMC9366546; doi:10.1001/jamanetworkopen.2022.25973)
Supplement: Supplement. — eTable 1. Landmark Regulatory Policies Concerning Drug Approvals in China, 2009-2020 eFigure 1. Identification of Sample Indications eBox 1. Key Words and Research Strategy to Identify Peer-Reviewed Publications of Drug Trials eTable 2. Outcome Variables Extracted From Trials eTable 3. Classification of Overall Survival (OS) Benefit eBox 2. Key Words and Research Strategy to Identify Peer-Reviewed Publications of Correlations Between Surrogate End Points and Overall Survival eTable 4. Sources of Information eFigure 2. Approval Year of Indications Supported Only by Single-Arm or Dose-Optimization Trials eFigure 3. Classification of Overall Survival (OS) Benefit for Cancer Indications Approved in China, by Approval Year eTable 5. Line of Therapy of Cancer Indications of Drugs Approved in China Only and Drugs Also Approved in the United States or the European Union eTable 6. Classification of Overall Survival and ESMO-MCBC Score of Cancer Drugs eTable 7. Magnitude of Documented Overall Survival (OS) Benefit for 68 Indications, by Cancer Type eTable 8. Results of Pivotal Trials Supporting 34 Indications With Documented Lack of Statistically Significant Overall Survival (OS) Benefit eTable 9. Primary End Point and Time From Enrollment to Approval of 13 Indications With Unknown Overall Survival Data by June 30, 2021 eTable 10. Results of Single-Arm or Dose-Optimization Trials Supporting Approvals by China National Medical Products Administration, 2005-2020 eTable 11. Published Surrogate Correlation Studies for Cancer Indications Without Documented Overall Survival Benefit References [file jamanetwopen-e2225973-s001.pdf]

Supplementary Online Content

Zhang Y, Naci H, Wagner AK, et al. Overall survival benefits of cancer drugs approved in China from 2005 to 2020. *JAMA Netw Open*. 2022;5(8):e2225973.
doi:10.1001/jamanetworkopen.2022.25973

eTable 1. Landmark Regulatory Policies Concerning Drug Approvals in China, 2009-2020
eFigure 1. Identification of Sample Indications
eBox 1. Key Words and Research Strategy to Identify Peer-Reviewed Publications of Drug Trials
eTable 2. Outcome Variables Extracted From Trials
eTable 3. Classification of Overall Survival (OS) Benefit
eBox 2. Key Words and Research Strategy to Identify Peer-Reviewed Publications of Correlations Between Surrogate End Points and Overall Survival
eTable 4. Sources of Information
eFigure 2. Approval Year of Indications Supported Only by Single-Arm or Dose-Optimization Trials
eFigure 3. Classification of Overall Survival (OS) Benefit for Cancer Indications Approved in China, by Approval Year
eTable 5. Line of Therapy of Cancer Indications of Drugs Approved in China Only and Drugs Also Approved in the United States or the European Union
eTable 6. Classification of Overall Survival and ESMO-MCBC Score of Cancer Drugs
eTable 7. Magnitude of Documented Overall Survival (OS) Benefit for 68 Indications, by Cancer Type
eTable 8. Results of Pivotal Trials Supporting 34 Indications With Documented Lack of Statistically Significant Overall Survival (OS) Benefit
eTable 9. Primary End Point and Time From Enrollment to Approval of 13 Indications With Unknown Overall Survival Data by June 30, 2021
eTable 10. Results of Single-Arm or Dose-Optimization Trials Supporting Approvals by China National Medical Products Administration, 2005-2020
eTable 11. Published Surrogate Correlation Studies for Cancer Indications Without Documented Overall Survival Benefit
References.

This supplementary material has been provided by the authors to give readers additional information about their work.

©2022 Zhang Y et al. *JAMA Network Open*.

Summary of regulatory policy changes in China, 2005-2020

eTable 1. Landmark Regulatory Policies Concerning Drug Approvals in China, 2009-2020

Effective Date	Issuing Organization	Policy Title	Main Policy Content
Jan, 2009	SFDA	Regulations on the Special Review for New Drug Registration	Put forward *Special Review* program, aiming to encourage new drug R&D. For conditions without effective treatments, such as AIDS, cancers, and rare diseases.
Feb, 2013	CFDA	Strengthening the Reform of Drug Review and Approval and Further Encouraging Innovation	To promote drug regulatory reform, improve review efficiency, and encourage R&D of novel drugs and generic drugs with clinical value.<br>Put forward principles of *Accelerated Review* and *Priority Review* programs.<br>Proposed to strengthen the quality management of drug clinical trials and encourage the development of drugs for children.
Aug, 2015	The State Council	Opinions of the State Council on Reform of the System for Evaluation, Review and Approval of Drugs and Medical Devices (No. 44 [2015])	Put forward the primary goals and missions of regulatory system reform, and measures to ensure its implementation.<br>Primary goals: to improve review quality, resolve drug registration backlog, improve quality of generic drugs, encourage new drug R&D, and improve review transparency.
Feb, 2016	CFDA	Resolving the Backlog of Drug Registration Applications and Implementing Priority Review and Approval	Implemented *Priority Review* program.<br>• Rolling review and shorter clock for review of marketing application<br>• Drugs for serious or life-threatening diseases, with early clinical evidence and surrogate endpoint that is reasonably likely to predict clinical benefit could get market authorization under *Conditional Approval*.<br>Certain drugs with clinically meaningful benefit and drugs intended to treat specific diseases were eligible for *Priority Review*, including:<br>• New drugs did not been approved in China or other countries, etc.;<br>• Drugs to treat AIDS, viral hepatitis, rare diseases, cancers, pediatric conditions, specific and frequent diseases of older adults, and others.

©2022 Zhang Y et al. *JAMA Network Open*.

Effective Date	Issuing Organization	Policy Title	Main Policy Content
Oct, 2017	General Office of the CPC Central Committee, and the General Office of the State Council	Opinions of the CPC Central Committee and the State Council on Deepening the Reform of the Evaluation and Approval Systems and Encouraging Innovation on Drugs and Medical Devices (No. 42 [2017])	Put forward the primary goals to strengthen the regulatory system reform on the basis of *No. 44 [2015]* and previous work: to encourage innovation, improve industry competitiveness, and meet public clinical needs.<br>Specific opinions:<br>• Reforming clinical trial management;<br>• Accelerating drug and medical devices review and approval;<br>• Encouraging drug innovation and the development of generic drugs;<br>• Strengthening the whole life cycle management of drugs and medical devices;<br>• Improving technical support capabilities;<br>• Strengthening relevant organization and implementation.
Dec, 2017	CFDA	Encouraging Drug Innovation and Implementing Priority Review and Approval	Revised *Priority Review* program, aiming to encourage drug innovation. A drug with clinically meaningful benefit, or a drug intended to treat specific diseases was eligible for *Priority Review* (revised):<br>• New drugs that did not launch in or outside China, and new drugs for which clinical trials were carried out by the National Clinical Medical Research Centre, and others<br>• Drugs to treat AIDS, viral hepatitis, rare diseases, cancers, pediatric conditions, specific and frequent diseases of older adults, and others;<br>• In the case of a major threat to public health, the drug registration application that has obtained a compulsory license will be given *Priority Review* and approval.<br>• Others.
Dec, 2017	CFDA	Publicly Solicit Comments on the *Technical Guidance for the Conditional Approval of Clinically*	Soliciting public comments for the *Technical Guidance for the Conditional Approval of Clinically Urgently Needed Drugs*, to accelerate the approval of urgently needed drugs with clinical value.<br>*Conditional Approval:*

©2022 Zhang Y et al. *JAMA Network Open*.

Effective Date	Issuing Organization	Policy Title	Main Policy Content
		Urgently Needed Drugs (Draft for Comments)	• A drug for serious or life-threatening diseases that have no available therapy;<br>• Approval based on surrogate endpoints or preliminary clinical trial evidence that is reasonably likely to predict clinical benefit.
Jul, 2018	NMPA	Announcement of the NMPA on Issuing the *Technical Guidance for Accepting Overseas Clinical Trial Data*	Implemented the opinions put forward in *No. 42 [2017]*, aiming to strengthen the guidance and standardization of the procedure of accepting overseas clinical trials.
Nov, 2018	NMPA	Notice on the Issuance of the First Batch of Clinically Urgently Needed Overseas New Drugs	Listed 40 drugs approved overseas, for which clinically urgently needs were identified in China, including 8 cancer drugs.
Mar, 2019	NMPA	Notice on the Issuance of the Second Batch of Clinically Urgently Needed Overseas New Drugs	Listed 26 drugs approved overseas for which clinically urgently needs were identified in China, including 2 cancer drugs.
Jul, 2020	NMPA	Announcement of the NMPA on Issuing the *Regulatory Review Procedures of Breakthrough Therapy (Trial)* and other three documents	Implemented *Breakthrough Therapy* designation, aiming to encourage R&D of drugs with clinically meaningful benefits.<br>*Breakthrough Therapy (pilot):*<br>• For a drug intended to treat a serious condition, where there is no available therapy, or the drug has an improved effect compared to available therapy;<br>• Preliminary clinical evidence that suggests substantial improvement over existing therapies.<br>Revised *Priority Review* and *Conditional Approval* program, aiming to accelerate approval of drugs with clinically meaningful benefits.<br>*Priority Review* (revised, pilot):

©2022 Zhang Y et al. *JAMA Network Open*.

Effective Date	Issuing Organization	Policy Title	Main Policy Content
			• Novel drugs for diseases for which there are clinically urgent needs, major infectious diseases and rare diseases;<br>• New varieties, dosage forms and specifications of childhood drugs;<br>• Vaccines urgently needed for disease prevention and control;<br>• Drugs designated as *Breakthrough Therapy*;<br>• Drugs eligible for *Conditional Approval*;<br>*Conditional Approval* (revised, pilot):<br>• Drugs for the treatment of serious life-threatening diseases for which there are no effective available therapies and with clinical trials that have confirmed efficacy and can predict the drugs' clinical value;<br>• Drugs that are urgently needed in public health, and that have clinical trials that have shown efficacy and can predict the clinical benefit of the drugs;<br>• Vaccines that are urgently needed in response to major public health emergencies or other vaccines that are determined to be urgently needed by the National Health Commission, whose benefits outweigh the risks after assessment.
Nov, 2020	NMPA	Notice on the Issuance of the Third Batch of Clinically Urgently Needed Overseas New Drugs	Listed 7 drugs approved overseas for which there are clinically urgently needs in China, including 2 cancer drugs.

Abbreviations: *CPC, the Communist Party of China; SFDA, State Food and Drug Administration; CFDA, China Food and Drug Administration; NMPA, National Medical Products Administration; R&D, research and development.*

©2022 Zhang Y et al. *JAMA Network Open*.

Methods

eFigure 1. Identification of Sample Indications

©2022 Zhang Y et al. *JAMA Network Open*.

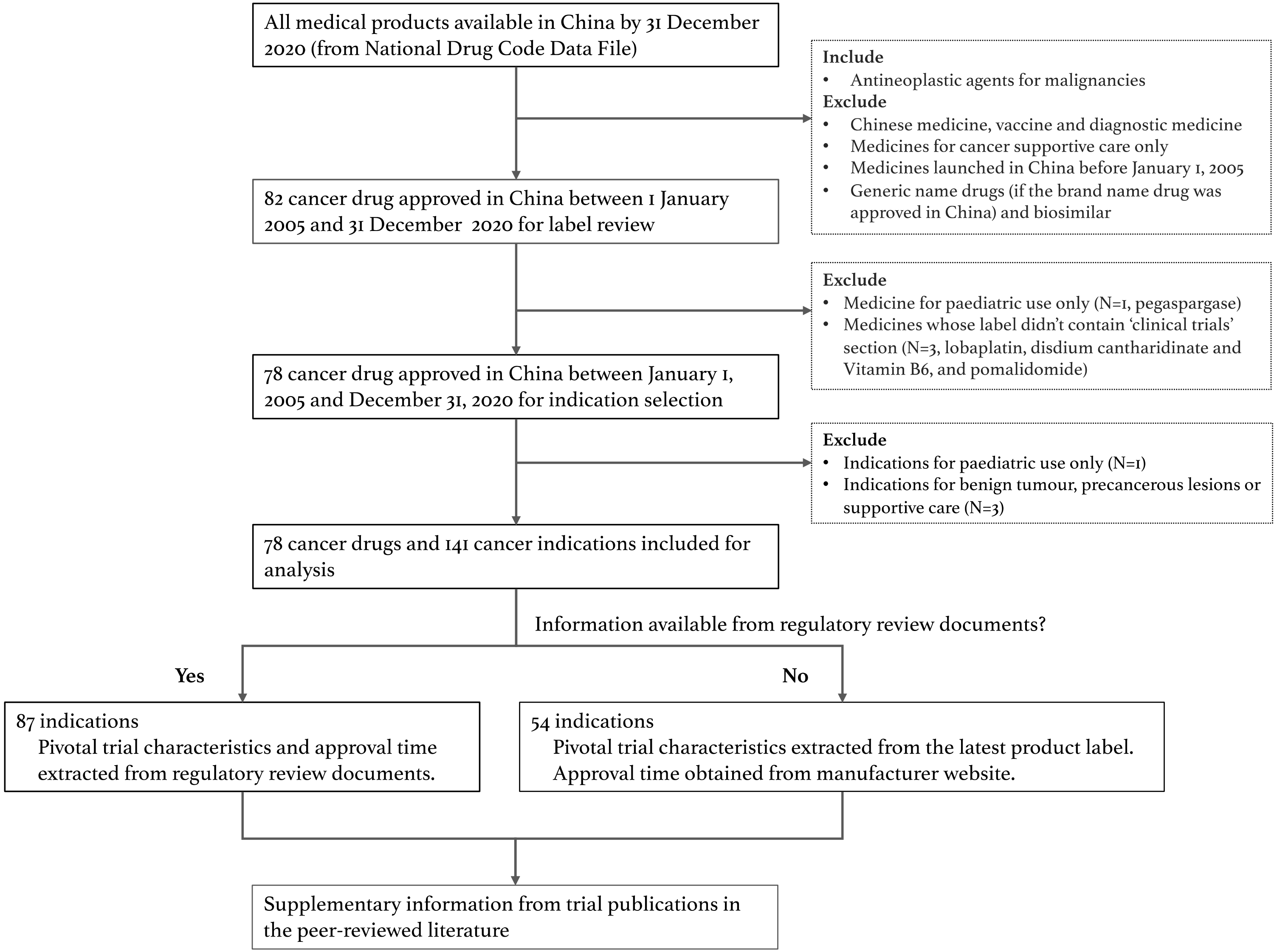

©2022 Zhang Y et al. *JAMA Network Open*.

eBox 1. Key Words and Research Strategy to Identify Peer-Reviewed Publications of Drug Trials

Key words

#1 NCT number

#2 CTR number

#3 Study name

#4 Generic name

#5 Approved indications (when necessary)

#6 "1900/1/1"[Date - Publication] : "2021/6/30"[Date - Publication]

Research strategies

PubMed: (#1 OR (#3 & #4 [Title/Abstract]) OR (#4 & #5 [Title/Abstract])) AND #6

CNKI: #2 OR #3 OR #4

Clinicaltrial.gov #1 OR #2 OR #3

Note: *CTR, Clinical Trial Registration; CNKI, China National Knowledge Infrastructure*.

©2022 Zhang Y et al. *JAMA Network Open*.

eTable 2. Outcome Variables Extracted From Trials

Endpoints (Abbreviation)	Definition
**Survival related endpoint**	
Overall survival (OS)	The time from randomization to death from any cause.
Progression-free survival (PFS)	The time from randomization to disease progression or death determined by Response Evaluation Criteria in Solid Tumors (RECIST) or other guidelines.
Disease-free survival (DFS)	The time from randomization to disease recurrence, new cancer occurrence, or death from any cause.
Relapse-free survival (RFS)	The time from randomization to disease recurrence or death from any cause.
**Tumor response**	
Objective response rate (ORR)	The percentage of patients who have complete response or partial response according to RECIST or other guidelines.
Disease control rate (DCR)	The percentage of patients who have complete response, partial response, stable disease or no evidence of disease according to RECIST guidelines or other criteria; also called clinical benefit rate.
**Response rate of hematologic malignancy**	
Major cytogenetic response (MCyR)	Complete (0 percent Ph-chromosome–positive cells in metaphase in bone marrow) or partial (1 to 35 percent Ph-chromosome–positive cells in metaphase).

©2022 Zhang Y et al. *JAMA Network Open*.

eTable 3. Classification of Overall Survival (OS) Benefit

Type	Example
**1. Documented OS benefit**	
1.1 Overall survival (OS) in the intervention group was statistically significantly longer than that in the comparison group in the pre-planned analysis	e.g., In COU-AA-301 (NCT00638690), pivotal trial of abiraterone (1st-line, metastatic castration-resistant prostate cancer), median OS was 15.8 months vs 11.2 months in abiraterone acetate group and placebo group (HR=0.74, p<0.0001).[1]
1.2 OS in the intervention group was statistically significantly longer than that in the comparison group in pre-planned analysis on subgroup consistent with the indication population	e.g., In LUX-Lung 6 (NCT01121393), pivotal trial of afatinib (1st-line, EGFR mutation-positive lung cancer), median OS was 23.1 months in the afatinib group and 23.5 months in the gemcitabine-cisplatin group (HR 0.93, p=0.61). However, in pre-planned analyses, overall survival was significantly longer for patients with del19-positive tumors in the afatinib group than in the chemotherapy group: 31.4 months vs 18.4 months (HR=0.64, p=0.023).[2]
1.3 OS in the intervention group was statistically significantly longer than that in the control group after adjusting for the crossover	e.g., In VEG105192 (NCT00334282), pivotal trial of pazopanib (1st-line and 2nd-line, advanced and/or metastatic renal cell carcinoma), the difference in final OS between pazopanib- and placebo-treated patients was not statistically significant (22.9 vs 20.5 months, HR=0.91, one-sided P = .224). In inverse probability of censoring weighted analyses, pazopanib decreased mortality (HR=0.504, two-sided P = .002).[3]
**2. Documented lack of OS benefit**	

©2022 Zhang Y et al. *JAMA Network Open*.

Type	Example
2.1 Final OS in the intervention group shows no statistically significant positive difference from or is less than in the comparison group	e.g., In ICOGEN (NCT01040780), pivotal trial of icotinib (1st-line, EGFR mutation lung cancer), there is no significant difference in median OS data between iconitib and gefitinib (13.3 vs 13.9 months, HR=1.02, p=0.57).[4]
2.2 If final OS data is not mature, the latest estimated OS data showed no statistically significant difference between the intervention group and the comparison group	e.g., In SPARTAN (NCT01946204), pivotal trial of apalutamide (1st-line, nonmetastatic castration-resistant prostate cancer), there is no significant difference in estimated median OS data (not reached vs 39.0 months, HR=0.70, p=0.07).[5]<br>e.g. In COMBI-AD (NCT01682083), pivotal trials of dabrafenib (adjuvant therapy, melanoma), the 3-year overall survival rate was 86% vs 77% (HR=0.57, p=0.0006), but this level of improvement did not cross the prespecified interim analysis boundary or P=0.000019.[6]
**3. Unknown OS benefit**	
3.1 None of the pivotal trials documented OS benefit data	e.g., In FESTnd Study (NCT02204644), pivotal trial of flumatinib (1st-line, newly diagnosed chronic-phase chronic myeloid leukemia), in the latest result published in Sep 2020, OS benefit between flumatinib and imatinib was not mentioned.[7]
3.2 OS data immature or not published by the end of observation	e.g., In NOVA (NCT01847274), pivotal trial of niraparib (maintenance therapy in platinum-sensitive, recurrent ovarian cancer), at the time of the database lock in latest publication, the results for the time until overall survival were not mature.[8]
**4. OS benefit not evaluable**	

©2022 Zhang Y et al. *JAMA Network Open*.

**Type**	**Example**
4.1 Indication supported by single arm trial only, that is, OS benefit not evaluable	e.g., Camrelizumab in relapsed or refractory classical Hodgkin lymphoma was approved on SHR-1210-II-204 (NCT03155425), a single-arm phase 2 study.[9]

©2022 Zhang Y et al. *JAMA Network Open*.

eBox 2. Key Words and Research Strategy to Identify Peer-Reviewed Publications of Correlations Between Surrogate End Points and Overall Survival

Key words

#1 Cancer site in specific setting

#2 Correlation OR Association OR Validation OR Relationship

#3 Surrogate endpoint in specific cancer site and cancer setting

#4 Overall survival

#5 Trial OR Studies

Research strategies in PubMed

(#1 [Title/Abstract]) AND (#2 [Title/Abstract]) AND (#3 [Title/Abstract]) & (#4 [Title/Abstract]) AND (#5 [Title/Abstract])

e.g., (advanced melanoma[title/abstract]) and ((correlation[title/abstract]) or (association[title/abstract]) or (validation[title/abstract]) or (relationship[title/abstract])) and ((progression-free survival[title/abstract]) or (response rate[title/abstract])) and (overall survival [title/abstract]) and ((trial[title/abstract]) or (studies[title/abstract]))

Note: *the search terms were similar with a previous study*

©2022 Zhang Y et al. *JAMA Network Open*.

Results

eTable 4. Sources of Information

Information source	No. Indication (%)
**Data sources for pivotal trials**	**141 (100)**
Regulatory review document [a]	87 (61.7)
Label [b]	54 (38.3)
**Data sources for approval time [c]**	
Regulatory review document	87 (61.7)
Label	19 (13.5)
Manufacture website	34 (24.1)
**Data sources for overall survival data**	
Regulatory review document	87 (61.7)
Label	6 (4.3)
Peer-reviewed publication as supplementary source [a]	128 (90.8)

Note:

[a] *By June 30, 2021*

[b] *By December 31, 2020*

[c] *All the data were crosschecked using a commercial dataset (http://db.pharmcube.com/database/drugde/main).*

©2022 Zhang Y et al. *JAMA Network Open*.

eFigure 2. Approval Year of Indications Supported Only by Single-Arm or Dose-Optimization Trials

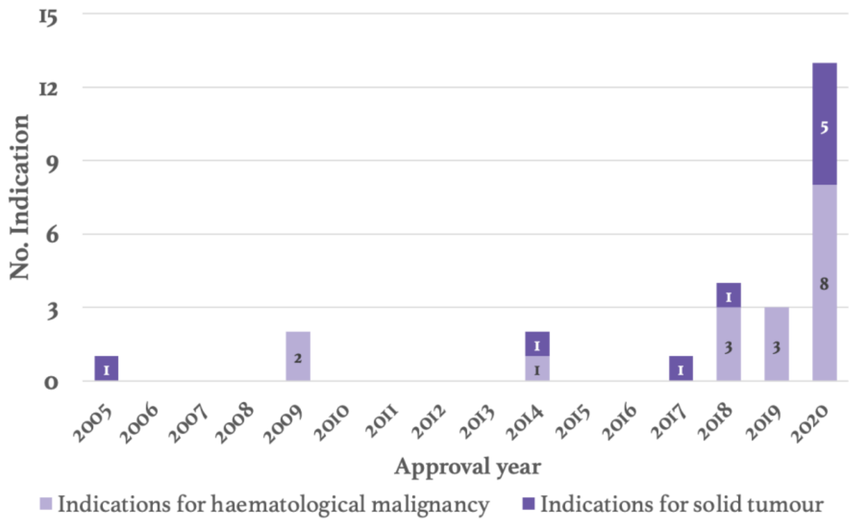

©2022 Zhang Y et al. *JAMA Network Open*.

eFigure 3. Classification of Overall Survival (OS) Benefit for Cancer Indications Approved in China, by Approval Year

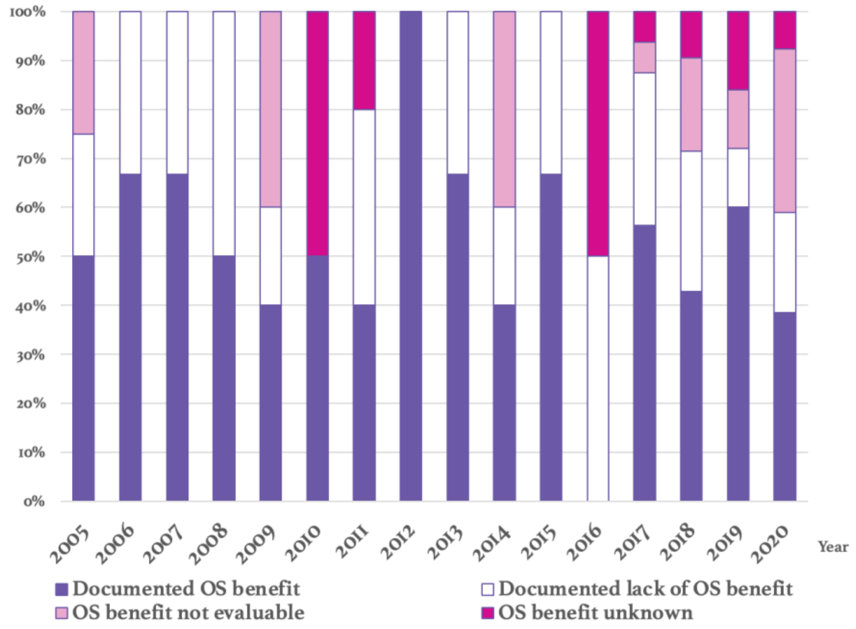

eTable 5. Line of Therapy of Cancer Indications of Drugs Approved in China Only and Drugs Also Approved in the United States or the European Union

Line of therapy	Indications, n (%)	Indications, n (%)	
		Of cancer drugs approved in China only [a]	Of cancer drugs also approved in the US or Europe[a]
**Total**	**141 (100)**	**30 (100)**	**111 (100)**
First-line therapy	67 (47.5)	8 (26.7)	59 (53.2)
Later therapy	67 (47.5)	22 (73.3)	45 (40.5)
Adjuvant/neoadjuvant therapy	7 (5.0)	0	7 (6.3)

[a] *By June 30, 2021; drug indications for more than one line of therapy were classified by the earliest line (neoadjuvant/adjuvant before first before later line).*

©2022 Zhang Y et al. *JAMA Network Open*.

eTable 6. Classification of Overall Survival and ESMO-MCBC Score of Cancer Drugs

Overall survival benefit type	Indication, n (%)	Applicable to ESMO-MCBS		
		Indications with ESMO-MCBS score, n (%, of all indications)		Indications with clinically meaningful benefit, n (%, of indications with ESMO-MCBS score)
**Total**	**141 (100)**	**66 (46.8)**		**38 (57.6)**
Documented OS benefit	68 (48.2)	42 (61.8)		29 (69.0)
Documented lack of OS benefit	34 (24.1)	18 (52.9)		8 (44.4)
OS benefit unknown	13 (9.2)	5 (38.5)		1 (20.0)
OS benefit not evaluable	26 (18.4)	1 (3.8)		0

Note: *European Society for Medical Oncology-Magnitude of Clinical Benefit Scale (ESMO-MCBS) scores were obtained from ESMO website in June 30, 2021 (https://www.esmo.org/guidelines/esmo-mcbs/esmo-mcbs-scorecards). ESMO-MCBS score A/B in the curative setting or 5/4 in the non-curative setting were considered clinically meaningful benefits. ESMO-MCBS scores are only available for solid tumor indications.*

©2022 Zhang Y et al. *JAMA Network Open*.

eTable 7. Magnitude of Documented Overall Survival (OS) Benefit for 68 Indications, by Cancer Type

No	Generic Name (China-only approval)	NMPA approved indication	Pivotal trial [reference]	Trial ID	Intervention	Comparison	OS data (month) [a]	OS benefit (month) [a]	HR (95% CI)	Note
**Lung**										
1	Afatinib	1st-line advanced or metastatic NSCLC with EGFR mutations	LUX-Lung 6[2]	NCT01121393	afatinib	gemcitabine + cisplatin	31.4 vs 18.4	13.0	0.64 (0.44-0.94)	del19-positive tumors
2	Afatinib	2nd-line advanced or metastatic NSCLC	LUX-Lung 8[10]	NCT01523587	afatinib	erlotinib	7.9 vs 6.8	1.1	0.81 (0.69-0.95)	
3	Alectinib	ALK mutation positive advanced or metastatic NSCLC	ALEX[11]	NCT02075840	alectinib	crizotinib	NR vs 57.4	NA	0.67 (0.46-0.98)	
4	Atezolizumab	1st-line ES-SCLC, in combination with carboplatin and etoposide	IMpower133[12]	NCT02763579	atezolizumab	placebo	12.3 vs 10.3	2.0	0.70 (0.54-0.91)	
5	Anlotinib (China-only approval)	3rd-line advanced or metastatic NSCLC	ALTER 0303[13]	NCT02388919	anlotinib	placebo	9.5 vs 6.4	3.1	0.70 (0.55-0.89)	
6	Anlotinib (China-only approval)	3rd-line progressed or relapse SCLC	ALTER 1202	NCT03059797	anlotinib	placebo	7.3 vs 4.9	2.4	0.53 (0.30-0.92)	Data obtained from label
7	Osimertinib	1st-line advanced or metastatic NSCLC with EGFR mutation (exon 19 deletion or L858R allele)	FLAURA[14]	NCT02296125	osimertinib	standard EGFR-TKI	38.6 vs 31.8	6.8	0.80 (0.64-1.00)	
8	Bevacizumab	1st-line, non-squamous NSCLC, in combination with platinum-based chemotherapy	BEYOND[15]	NCT01364012	Bevacizumab + cisplatin + gemcitabine	Placebo + cisplatin + gemcitabine	24.3 vs 17.7	6.6	0.68 (0.50-0.93)	
9	Dacomitinib	1st-line, advanced or metastatic NSCLC with EGFR mutation (exon 19 deletion or L858R allele)	ARCHER 1050[16]	NCT01774721	dacomitinib	gefitinib	34.1 vs 27.0	7.1	0.75 (0.59-0.95)	

©2022 Zhang Y et al. *JAMA Network Open*.

No	Generic Name (China-only approval)	NMPA approved indication	Pivotal trial [reference]	Trial ID	Intervention	Comparison	OS data (month) [a]	OS benefit (month) [a]	HR (95% CI)	Note
10	Durvalumab	Maintenance treatment, unresectable stage III NSCLC following platinum-based chemotherapy and radiation therapy	PACIFIC[17]	NCT02125461	durvalumab	placebo	NR vs 29.1	NA	0.69 (0.55-0.86)	
11	Erlotinib	Maintenance treatment, locally advanced or metastatic NSCLC, following 4 cycles platinum-based chemotherapy	SATURN[18]	NCT00556712	erlotinib	placebo	12.0 vs 11.0	1.0	0.81 (0.70-0.95)	
12	Erlotinib	2nd line, locally advanced or metastatic NSCLC	BR.21	NCT00036647	erlotinib	placebo	6.7 vs 4.7	2.0	0.70 (0.58-0.85)	
13	Crizotinib	ALK mutation positive locally advanced or metastatic NSCLC	PROFILE 1014[19]	NCT01154140	crizotinib	pemetrexed + cisplatin/carbo platin	NR vs 47.5	NA	0.35 (0.08-0.72)	adjusted for crossover
14	Nivolumab	2nd-line, EGFR and ALK mutation negative, locally advanced or metastatic NSCLC, after platinum-based chemotherapy	CheckMate 017[20]	NCT01642004	nivolumab	docetaxel	9.2 vs 6.0	3.2	0.59 (0.44-0.79)	
15	Pembrolizumab	1st-line, metastatic non-squamous NSCLC with EGFR and ALK mutation negative, in combination with pemetrexed and platinum-based chemotherapy	KEYNOTE-189[21]	NCT02578680	pembrolizumab	placebo	22.0 vs 10.7	11.3	0.56 (0.45-0.70)	
16	Pembrolizumab	1st-line, locally advanced or metastatic NSCLC expressing	KEYNOTE-042 （China study）[22]	NCT03850444	pembrolizumab	chemotherapy	20.2 vs 13.5	6.7	0.67 (0.50-0.89)	

©2022 Zhang Y et al. *JAMA Network Open*.

No	Generic Name (China-only approval)	NMPA approved indication	Pivotal trial [reference]	Trial ID	Intervention	Comparison	OS data (month) [a]	OS benefit (month) [a]	HR (95% CI)	Note
		PD-L1 TPS≥1, with EGFR and ALK mutation negative								
17	Pembrolizumab	1st-line, metastatic NSCLC, in combination with carboplatin and paclitaxel	KEYNOTE-407[23]	NCT02775435	pembrolizumab	placebo	17.1 vs 11.6	5.5	0.71 (0.58-0.88)	
18	Pemetrexed	1st-line, locally advanced or metastatic NSCLC, in combination with cisplatin	H3E-MC-JMDB[24]	NCT00087711	pemetrexed+ cisplatin	gemcitabine+ cisplatin	11.8 vs 10.4	1.4	0.81 (0.70-0.94)	
19	Pemetrexed	Maintenance treatment, locally advanced or metastatic non-squamous NSCLC, following 4 cycle platinum-based chemotherapy	H3E-MC-JMEN[25]	NCT00102804	pemetrexed+ best supportive care	placebo+ best supportive care	13.4 vs 10.6	2.8	0.79 (0.65-0.95)	
20	Recombinant Human Endostatin (China-only approval)	1st-line, stage III/IV NSCLC, in combination with vinorelbine and cisplatin	A Phase 3 study	NA	Recombinant Human Endostatin+ vinblastine+ cisplatin	vinblastine+ cisplatin	14.9 vs 9.9	5.0	Not mentioned	Data obtained from label
**Prostate**										
21	Abiraterone	mCRPC	COU-AA-301[1]	NCT00638690	abiraterone acetate + prednisone	placebo + prednisone	15.8 vs 11.2	4.6	0.74 (0.64-0.86)	
22	Abiraterone	Newly diagnosed mHSPC	LATITUDE[26]	NCT01715285	androgen-deprivation therapy + abiraterone acetate + prednisone	androgen-deprivation therapy + dual placebos	53.3 vs 36.5	16.8	0.66 (0.56-0.78)	

©2022 Zhang Y et al. *JAMA Network Open*.

No	Generic Name (China-only approval)	NMPA approved indication	Pivotal trial [reference]	Trial ID	Intervention	Comparison	OS data (month) [a]	OS benefit (month) [a]	HR (95% CI)	Note
23	Apalutamide	metastatic castration-sensitive prostate cancer	TITAN[27]	NCT02489318	apalutamide+ androgen-deprivation therapy	placebo++and rogen-deprivation therapy	NR vs NR	NA	0.67 (0.51-0.89)	
24	Enzalutamide	mCRPC	PREVAIL[28]	NCT01212991	enzalutamide	placebo	36.0 vs 31.0	5.0	0.83 (0.75-0.93)	
25	Enzalutamide	Non-metastatic CRPC with a high risk of metastasis	PROSPER[29]	NCT02003924	enzalutamide	placebo	67.0 vs 56.3	10.7	0.73 (0.61-0.89)	
26	Radium (223Ra) Dichloride	mCRPC with symptomatic bone metastasis and no visceral metastasis	ALSYMPCA[30]	NCT00699751	radium-223	placebo	14.9 vs 11.3	3.6	0.70 (0.58-0.83)	
**Colon and rectum**										
27	Bevacizumab	mCRC, in combination with fluorouracil-based chemotherapy	AVF2107g[31]	NCT00109070	bevacizumab + IFL	placebo + IFL	20.3 vs 15.6	4.7	0.66	
28	Fruquintinib (China-only approval)	3rd line, mCRC	FRESCO[32]	NCT02314819	fruquintinib	placebo	9.3 vs 6.6	2.7	0.65 (0.51-0.83)	
29	Trifluridine and Tipiracil	3rd line, mCRC	RECOURSE[33]	NCT01607957	trifluridine/tipir acil	placebo	7.1 vs 5.3	1.8	0.68 (0.58-0.81)	
30	Regorafenib	3rd line, mCRC	CONCUR[34]	NCT01584830	regorafenib	placebo	8.8 vs 6.3	2.5	0.55 (0.40-0.77)	
31	Cetuximab	1st-line, RAS wild-type mCRC, in combination with FOLFOX or FOLFIRI	TAILOR	NCT01228734	cetuximab+FO LFOX-4	FOLFOX-4	20.8 vs 16.5	4.3	0.76 (0.61-0.95)	Data obtained from regulatory review document
**Multiple myeloma**										
32	Lenalidomide	untreated and unsuitable for transplantation multiple myeloma, in	FIRST[35]	NCT00689936	continuous lenalidomide+ dexamethasone	melphalan+ prednisone+ thalidomide	59.1 vs 49.1	10.0	0.78 (0.67-0.92)	

©2022 Zhang Y et al. *JAMA Network Open*.

No	Generic Name (China-only approval)	NMPA approved indication	Pivotal trial [reference]	Trial ID	Intervention	Comparison	OS data (month) [a]	OS benefit (month) [a]	HR (95% CI)	Note
		combination with dexamethasone								
33	Lenalidomide	2nd-line, multiple myeloma, in combination with dexamethasone	MM-009[36]	NCT00056160	lenalidomide	placebo	29.6 vs 20.2	9.4	0.44 (0.30-0.65)	
34	Bortezomib	multiple myeloma relapsed after at least one treatment	APEX[37]	NCT00048230	bortezomib	dexamethasone	29.8 vs 23.7	6.1	0.77	
35	Bortezomib	untreated and unsuitable for high-dose chemotherapy and myelosuppression multiple myeloma, in combination with melphalan and prednisone	VISTA[38 39]	NCT00111319	bortezomib+ melphalan+ prednisone	melphalan+ prednisone	56.4 vs 43.1	13.3	0.70 (0.57-0.85)	
36	Ixazomib	2nd-line, multiple myeloma, in combination with lenalidomide and dexamethasone	TOURMALINE-MM1 Chinese expansion[40]	NCT01564537	ixazomib+ lenalidomide+ dexamethasone	placebo+ lenalidomide+ dexamethasone	25.8 vs 15.8	10.0	0.42 (0.24-0.73)	
**Breast**										
37	Eribulin	3rd-line advanced or metastatic breast cancer after anthracycline and taxane-based chemotherapy	EMBRACE[41]	NCT00388726	eribulin	treatment of physician's choice	13.1 vs 10.6	2.5	0.81 (0.66-0.99)	
38	Pyrotinib (China-only approval)	2nd line relapsed or metastatic HER2 positive breast cancer after anthracycline and taxane-based chemotherapy, in	HR-BLTN-I/II-MBC	NCT02422199	pyrotinib + capecitabine	lapatinib + capecitabine	NR vs 16.2	NA	0.36 (0.16-0.82)	Data obtained from regulatory review document

©2022 Zhang Y et al. *JAMA Network Open*.

No	Generic Name (China-only approval)	NMPA approved indication	Pivotal trial [reference]	Trial ID	Intervention	Comparison	OS data (month) [a]	OS benefit (month) [a]	HR (95% CI)	Note
		combination with capecitabine								
39	Pertuzumab	metastatic or unresectable locally relapsed HER2-positive breast cancer, in combination with trastuzumab and docetaxel	CLEOPATRA[42]	NCT00567190	pertuzumab +trastuzumab + docetaxel	placebo+ trastuzumab+ docetaxel	57.1 vs 40.8	16.3	0.69 (0.58-0.82)	
40	Abemaciclib	HR positive, HER2-negative advanced or metastatic breast cancer with disease progression following endocrine therapy, in combination with fulvestrant	MONARCH 2	NCT02107703	abemaciclib + fulvestrant	placebo + fulvestrant	46.7 vs 37.3	9.4	0.757 (0.606-0.945)	
**Stomach**										
41	Apatinib (China-only approval)	3rd-line gastric cancer	A Phase 2 study[43]	NCT00970138	apatinib 850 mg once daily or 425mg twice daily	placebo	4.8 vs 4.3 vs 2.5	2.3 (850mg); 1.8 (425mg)	0.51 (0.32-0.83)	850mg group
42	Nivolumab	3rd line, advanced or relapsed gastric or gastroesophageal junction cancer	ONO-4538-12[44]	NCT02267343	nivolumab	placebo	5.3 vs 4.1	1.2	0.63 (0.51-0.78)	
43	Tegafur, Gimeracil and Oteracil Potassium	unresectable locally advanced or metastatic gastric cancer	SC-101	NCT00202969	S-1+cisplatin	fluorouracil+ cisplatin	14.4 vs 10.3	4.1	Not mentioned	Data obtained from label
**Head and neck**										
44	Nivolumab	2nd-line, relapsed or refractory HNSCC expressing PD-L1 TPS≥1, with disease	CheckMate 141	NCT02105636	nivolumab	standard, single-agent systemic therapy	12.5 vs 5.9	6.6	0.38 (0.19-0.77)	Asian subgroup, data obtained

©2022 Zhang Y et al. *JAMA Network Open*.

No	Generic Name (China-only approval)	NMPA approved indication	Pivotal trial [reference]	Trial ID	Intervention	Comparison	OS data (month) [a]	OS benefit (month) [a]	HR (95% CI)	Note
		progression after or during platinum-containing regimens				(methotrexate, docetaxel, or cetuximab)				from regulatory review document
45	Pembrolizumab	1st-line, metastatic or unresectable relapsed HNSCC expressing PD-L1 CPS ≥1	KEYNOTE-048[45]	NCT02358031	pembrolizumab + platinum+ 5-fluorouracil	cetuximab+ platinum+ 5-fluorouracil	14.7 vs 11.0	3.7	0.60 (0.45-0.82)	
46	Cetuximab	1st-line, recurrent and/or metastatic HNSCC, in combination with platinum and fluorouracil chemotherapy	EXTREME[46]	NCT00122460	cetuximab+ cisplatin/carboplatin+ fluorouracil	cisplatin/carboplatin+ fluorouracil	10.1 vs 7.4	2.7	0.80 (0.64-0.99)	
**Leukemia**										
47	Azacitidine	CMML	AZA PH GL 2003 CL 001[47]	NCT00071799	azacitidine	conventional care	24.5 vs 15.0	9.5	0.58 (0.43-0.77)	
48	Ibrutinib	chronic lymphocytic leukemia/small lymphocytic lymphoma	RESONATE[48]	NCT01578707	ibrutinib	ofatumumab	67.7 vs 65.1	2.6	0.24 (0.11-0.55)	
49	Blinatumomab	relapsed or refractory precursor B-cell acute lymphoblastic leukemia	TOWER[49]	NCT02013167	blinatumomab	standard-of-care chemotherapy	7.7 vs 4.0	3.7	0.71 (0.55-0.93)	
**Melanoma**										
50	Dabrafenib	unresectable or metastatic melanoma with BRAF V600E or V600K mutations, in combination with trametinib	COMBI-d[50]	NCT01584648	dabrafenib + trametinib	dabrafenib + placebo	25.1 vs 18.7	6.4	0.71 (0.55-0.92)	

©2022 Zhang Y et al. *JAMA Network Open*.

No	Generic Name (China-only approval)	NMPA approved indication	Pivotal trial [reference]	Trial ID	Intervention	Comparison	OS data (month) [a]	OS benefit (month) [a]	HR (95% CI)	Note
51	Trametinib	unresectable or metastatic melanoma with BRAF V600E or V600K mutations, in combination with dabrafenib	COMBI-d[50]	NCT01584648	dabrafenib+ trametinib	dabrafenib+ placebo	25.1 vs 18.7	6.4	0.71 (0.55-0.92)	
52	Vemurafenib	unresectable or metastatic melanoma with BRAF V600 mutations	BRIM-3[51]	NCT01006980	vemurafenib	dacarbazine	13.6 vs 10.3	3.3	0.81 (0.68-0.96)	
**Liver**										
53	Apatinib (China-only approval)	advanced HCC failed or untolerated to at least first-line systemic treatment	AHELP[52]	NCT02329860	apatinib	placebo	8.7 vs 6.8	1.9	0.79 (0.62-1.00)	
54	Atezolizumab	unresectable HCC without previous systemic treatment, in combination with bevacizumab	IMbrave150[53]	NCT03434379	atezolizumab+ bevacizumab	sorafenib	NR vs 13.2	NA	0.58 (0.42-0.79)	
55	Regorafenib	HCC who has been previously treated with sorafenib	RESORCE[34]	NCT01774344	regorafenib	placebo	10.6 vs 7.8	2.8	0.63 (0.50-0.79)	
56	Sorafenib	unresectable or metastatic HCC	SHARP[54]	NCT00105443	sorafenib	placebo	10.7 vs 7.9	2.8	0.69 (0.55-0.87)	
**Esophagus**										
57	Camrelizumab (China-only approval)	2nd-line, locally advanced or metastatic esophageal squamous cell carcinoma	ESCORT[55]	NCT03099382	camrelizumab	investigator's choice of chemotherapy	8.3 vs 6.2	2.1	0.71 (0.57-0.87)	
58	Pembrolizumab	2nd-line, locally advanced or metastatic esophageal squamous	KEYNOTE-181 (China expansion study)	NCT03933449	pembrolizumab	investigator's choice of paclitaxel,	12.0 vs 5.3	6.7	0.34 (0.17-0.69)	Data obtained from

©2022 Zhang Y et al. *JAMA Network Open*.

No	Generic Name (China-only approval)	NMPA approved indication	Pivotal trial [reference]	Trial ID	Intervention	Comparison	OS data (month) [a]	OS benefit (month) [a]	HR (95% CI)	Note
		cell carcinoma expressing PD-L1 CPS ≥1				docetaxel, or irinotecan				Review Document
**Ovary**										
59	Olaparib	Maintenance treatment, recurrent epithelial ovarian, fallopian tube or primary peritoneal cancer, who are in complete or partial response to platinum-based chemotherapy	SOLO2 (D0816C00002)[56]	NCT01874353	olaparib	placebo	51.7 vs 35.4	16.3	0.56 (0.35-0.97)	adjust for subsequent PARP inhibitor therapy in the placebo group
60	Niraparib	Maintenance treatment, advanced epithelial ovarian cancer, fallopian tube cancer or primary peritoneal cancer, after platinum-based chemotherapy	PRIMA[57]	NCT02655016	niraparib	placebo	81% vs 59%	22%	0.51 (0.27-0.97)	24-month OS rate
**Lymphoma**										
61	Bortezomib	untreated and unsuitable for hematopoietic stem cell transplantation MCL, in combination with rituximab, cyclophosphamide, doxorubicin, and prednisone	LYM-3002[58]	NCT00722137	VR-CAP (rituximab+ cyclophosphamide+ doxorubicin+ bortezomib+ prednisone)	R-CHOP (rituximab+ cyclophosphamide+ doxorubicin+ vincristine+ prednisone)	90.7 vs 55.7	35.0	0.66 (0.51-0.85)	
62	Lenalidomide	previously treated follicular lymphoma, in combination with a rituximab product	AUGMENT	NCT01938001	lenalidomide+ rituximab	placebo+ rituximab	Estimated 2-year OS% 95% vs 86%	9%	0.45 (0.22-0.92)	Data obtained from regulatory review document

©2022 Zhang Y et al. *JAMA Network Open*.

No	Generic Name (China-only approval)	NMPA approved indication	Pivotal trial [reference]	Trial ID	Intervention	Comparison	OS data (month) [a]	OS benefit (month) [a]	HR (95% CI)	Note
**Brain**										
63	Temozolomide	1st-line, newly diagnosed glioblastoma multiforme, in combination with radiotherapy, and then as maintenance therapy	EORTC-NCIC[59]	NCT00006353	temozolomide+ radiotherapy	radiotherapy	14.6 vs 12.1	2.5	0.63 (0.52-0.75)	
**Kidney**										
64	Pazopanib	1st-line or 2nd-line advanced RCC	VEG105192[3]	NCT00334282	pazopanib	placebo	22.9 vs 20.5	2.4	0.50 (0.32-0.76)	adjusted for crossover
**Mesothelioma**										
65	Pemetrexed	unresectable malignant pleural mesothelioma, in combination of cisplatin	JMCH[60]	NCT00005636	pemetrexed disodium+ cisplatin	cisplatin	12.1 vs 9.3	2.8	0.77 (0.61-0.96)	
**Nasopharynx**										
66	Nimotuzumab (China-only approval)	stage III/IV nasopharyngeal cancer with EGFR mutation, in combination with radiotherapy	A Phase 2 study	NA	nimotuzumab+ radiation	radiation	36-month OS % 84.3% vs 77.6%	6.7%	NA	Data obtained from label; mean survival month: 35.8 vs 32.2
**Other**										
67	Sunitinib	progressive, well-differentiated pNET, with unresectable locally advanced or metastatic disease	A6181111[61 62]	NCT00428597	sunitinib+ best supportive care	placebo+ best supportive care	38.6 vs 16.3	22.3	0.40 (0.23-0.71)	Censoring at crossover
68	Sunitinib	GIST, after disease progression on or intolerance to imatinib mesylate	A6181004[63]	NCT00075218	sunitinib	placebo	NR vs NR	NA	0.49 (0.29-0.83)	

©2022 Zhang Y et al. *JAMA Network Open*.

Abbreviation: *HR, hazard ratio; NR, not reached; NA, not applicable; mCRPC, metastatic castration-resistant prostate cancer; mHSPC, high-risk metastatic endocrine therapy-sensitive prostate cancer; NSCLC, non-small cell lung cancer; EGFR, epidermal growth factor receptor; ALK, anaplastic lymphoma kinase; SCLC, small cell lung cancer; ES-SCLC, extensive-stage small cell lung cancer; mCRC, metastatic colorectal cancer; IFL, irinotecan, bolus fluorouracil, and leucovorin; PD-1, programmed cell death receptor-1; HNSCC, head and neck squamous cell cancer; TPS, tumor proportion score; CPS, combined positive score; RCC, renal cell carcinoma; MCL, mantle cell lymphoma; HCC, hepatocellular carcinoma; GIST, gastrointestinal stromal tumor; pNET, pancreatic neuroendocrine tumors; FOLFOX, leucovorin, fluorouracil, and oxaliplatin; FOLFIRI, leucovorin, fluorouracil, and irinotecan; HR, hormone receptor; HER2, human epidermal growth factor receptor 2.*

Note: *for indications with more than one pivotal trial showing overall survival benefit, the maximum survival gain was extracted for analysis.*

[a] *OS benefit measured in months unless otherwise indicated.*

©2022 Zhang Y et al. *JAMA Network Open*.

eTable 8. Results of Pivotal Trials Supporting 34 Indications With Documented Lack of Statistically Significant Overall Survival (OS) Benefit

No	Generic Name (China-only approval)	NMPA approved indication	Pivotal trial [reference]	Trial ID	Intervention	Comparison	Primary Endpoint	Primary Endpoint Results (month, HR [95%CI])	OS data (month) [a]	OS HR (95% CI)	Note
**Lung**											
1	Camrelizumab (China-only approval)	1st-line, unresectable locally advanced or metastatic non-squamous NSCLC with EGFR and ALK mutation negative, in combination with pemetrexed and carboplatin	CameL[64]	NCT03134872	camrelizumab+ carboplatin+ pemetrexed	carboplatin+ pemetrexed	PFS	11.3 vs 8.3 HR=0.60 (0.45-0.79)	NR vs 20.9	0.73 (0.53-1.02)	
2	Ceritinib	1st-line, locally advanced or metastatic NSCLC with ALK mutation positive	ASCEND-4[65]	NCT01828099	ceritinib	chemotherapy	PFS	16.6 vs 8.1 HR=0.55 (0.42-0.73)	NR vs 26.2	0.73 (0.50-1.08)	
3	Ceritinib	2nd-line, locally advanced or metastatic NSCLC with ALK mutation positive	ASCEND-5[66]	NCT01828112	ceritinib	chemotherapy (pemetrexed/docetaxel)	PFS	5.4 vs 1.6 HR=0.49 (0.36-0.67)	18.1 vs 20.1	1.0 (0.67-1.49)	
4	Erlotinib	1st-line, maintenance or 2nd line, locally advanced or metastatic NSCLC with	ENSURE[67]	NCT01342965	erlotinib	gemcitabine+ cisplatin	PFS	11.0 vs 5.5 HR=0.34 (0.22-0.51)	26.3 vs 25.5	0.91 (0.63-1.31)	

©2022 Zhang Y et al. *JAMA Network Open*.

No	Generic Name (China-only approval)	NMPA approved indication	Pivotal trial [reference]	Trial ID	Intervention	Comparison	Primary Endpoint	Primary Endpoint Results (month, HR [95%CI])	OS data (month) [a]	OS HR (95% CI)	Note
		EGFR mutation positive									
			EURTAC[68]	NCT00446225	erlotinib	standard chemotherapy	PFS	9.7 vs 5.2 HR=0.37 (0.25-0.54)	19.3 vs 19.5	1.04 (0.65-1.68)	
			OPTIMAL[69 70]	NCT00874419	erlotinib	gemcitabine+ carboplatin	PFS	13.1 vs 4.6 HR=0.16 (0.10-0.26)	22.8 vs 27.2	1.19 (0.83-1.71)	
5	Icotinib (China-only approval)	1st-line, locally advanced or metastatic NSCLC with EGFR mutation	ICOGEN[4]	NCT01040780	icotinib	gefitinib	PFS	4.6 vs 3.4 HR=0.84 (0.67-1.05)	13.3 vs 13.9	1.02 (0.82-1.27)	
6	Osimertinib	2nd-line, locally advanced or metastatic NSCLC with EGFR T790M mutation positive	AURA3[71 72]	NCT02151981	osimertinib	platinum+ pemetrexed	PFS	10.1 vs 4.4 HR=0.30 (0.23-0.41)	26.8 vs 22.5	0.87 (0.67-1.12)	
7	Pemetrexed	2nd-line, locally advanced or metastatic non-squamous NSCLC	H3E-MC-JMID[73]	NCT00391274	pemetrexed	docetaxel	OS	11.7 vs 12.2 HR=1.14 (0.78-1.68)	11.7 vs 12.2	1.14 (0.78-1.68)	
			JMEI[74]	NCT00004881	pemetrexed	docetaxel	OS	8.3 vs 7.9 HR=0.99 (0.82-1.20)	8.3 vs 7.9	0.99 (0.82-1.20)	
**Breast**											
8	Abemaciclib	initial endocrine-based therapy, locally advanced or metastatic breast cancer with HR positive,	MONARCH 3[75 76]	NCT02246621	abemaciclib+ non-steroidal aromatase inhibitor	placebo+ non-steroidal aromatase inhibitor	PFS	28.2 vs 14.8 HR=0.54 (0.42-0.70)	NR vs NR	1.06 (0.68-1.63)	Data obtained from label

©2022 Zhang Y et al. *JAMA Network Open*.

No	Generic Name (China-only approval)	NMPA approved indication	Pivotal trial [reference]	Trial ID	Intervention	Comparison	Primary Endpoint	Primary Endpoint Results (month, HR [95%CI])	OS data (month) [a]	OS HR (95% CI)	Note
		HER2 negative, in combination with an aromatase inhibitor									
			MONARCH plus Cohort A[77]	NCT02763566	abemaciclib+ non- steroidal aromatase inhibitor	placebo+ non-steroidal aromatase inhibitor	PFS	NR vs 14.7 HR=0.50 (0.35-0.72)	NR vs NR	Not reported	
9	Lapatinib	2nd-line, HER2 positive advanced or metastatic breast cancer, in combination with capecitabine	EGF100151[78 79]	NCT00078572	lapatinib+ capecitabine	capecitabine	TTP	6.2 vs 4.3 HR=0.57 (0.43-0.77)	18.8 vs 16.2	0.87 (0.71-1.08)	
10	Pertuzumab	adjuvant therapy HER2-positive early breast cancer at high risk of recurrence, in combination with trastuzumab and chemotherapy	APHINITY[80 81]	NCT01358877	pertuzumab+ standard adjuvant chemotherapy+ trastuzumab	placebo+ standard adjuvant chemotherapy + trastuzumab	3-year IDFS	92.3% vs 90.6% HR=0.81 (0.66-1.00)	6-year OS% 95% vs 94%	0.81 (0.66-1.00)	
11	Trastuzumab Emtansine	adjuvant therapy, residual invasive HER2-positive early breast cancer, after taxanes plus trastuzumab based neoadjuvant therapy	KATHERINE[82]	NCT01772472	trastuzumab emtansine	trastuzumab	DFS	88.3% vs 77.0% HR=0.50 (0.39-0.64)	NR vs NR	0.70 (0.47–1.05)	
**Kidney**											
12	Axitinib	advanced RCC after failure of	A4061051[83 84]	NCT00920816	axitinib	sorafenib	PFS	6.5 vs 4.8 HR=0.73	21.7 vs 23.3	1.00 (0.73-1.36)	

©2022 Zhang Y et al. *JAMA Network Open*.

No	Generic Name (China-only approval)	NMPA approved indication	Pivotal trial [reference]	Trial ID	Intervention	Comparison	Primary Endpoint	Primary Endpoint Results (month, HR [95%CI])	OS data (month) [a]	OS HR (95% CI)	Note
		tyrosine kinase inhibitor or cytokine therapy						(0.51-1.06)			
			AXIS[85 86]	NCT00678392	axitinib	sorafenib	PFS	8.3 vs 5.7 HR=0.66 (0.55-0.78)	20.1 vs 19.2	0.97 (0.80-1.17)	
13	Sunitinib	unresectable advanced RCC	A6181034[87]	NCT00083889	sunitinib	interferon alfa	PFS	11.0 vs 5.0 HR=0.42 (0.32-0.54)	28.7 vs 23.7	0.82 (0.67-1.00)	Data obtained from label
14	Sorafenib	unresectable advanced RCC	TARGET[88 90]	NCT00073307	sorafenib	placebo	PFS	5.5 vs 2.8 HR=0.44 (0.35-0.55)	17.8 vs 15.2	0.88 (0.74-1.04)	
15	Everolimus	advanced RCC, after failure of sunitinib or sorafenib therapy	RECORD1[89 90]	NCT00410124	everolimus	placebo	PFS	4.0 vs 1.9 HR=0.30 (0.22-0.40)	14.8 vs 14.4	0.87 (0.65-1.15)	
**Melanoma**											
16	Dabrafenib	adjuvant therapy, melanoma with BRAF V600 mutations, in combination with trametinib	COMBI-AD[91]	NCT01682083	dabrafenib+ trametinib	placebo	RFS	NR vs 16.6 HR=0.47 (0.39-0.58)	NR vs NR	0.57 (0.42-0.79)	Did not meet protocol-specified analysis boundary

©2022 Zhang Y et al. *JAMA Network Open*.

No	Generic Name (China-only approval)	NMPA approved indication	Pivotal trial [reference]	Trial ID	Intervention	Comparison	Primary Endpoint	Primary Endpoint Results (month, HR [95%CI])	OS data (month) [a]	OS HR (95% CI)	Note
17	Pembrolizumab	2nd-line, unresectable or metastatic melanoma	KEYNOTE-002[92 93]	NCT01704287	pembrolizumab 2 mg/kg<br>pembrolizumab 10 mg/kg	investigator-choice chemotherapy	PFS	2.9 vs 2.7 HR=0.58 (0.46-0.73)<br>2.9 vs 2.7 HR=0.47 (0.37-0.60)	13.4 vs 11.0<br>14.7 vs 11.0	0.86 (0.67-1.10)<br>0.74 (0.57-0.96)	Did not meet protocol-specified boundary
18	Trametinib	adjuvant therapy, melanoma with BRAF V600 mutations, in combination with dabrafenib	COMBI-AD[91]	NCT01682083	dabrafenib+ trametinib	placebo	RFS	NR vs 16.6 HR=0.47 (0.39-0.58)	3-year OS% 86% vs 77%	0.57 (0.42-0.79)	Level of improvement did not cross the prespecified interim analysis boundary
**Brain**											
19	Bevacizumab	recurrent glioblastoma	AVF3708g[94]	NCT00345163	bevacizumab+ irinotecan	bevacizumab	6-month PFS rate	50.3% vs 42.6% HR not mentioned	9.3 vs 8.8	not reported	
			EORTC26101[95]	NCT01290939	bevacizumab+ lomustine	lomustine	OS	9.1 vs 8.6 HR=0.95 (0.74-1.21)	9.1 vs 8.6	0.95 (0.74-1.21)	

©2022 Zhang Y et al. *JAMA Network Open*.

No	Generic Name (China-only approval)	NMPA approved indication	Pivotal trial [reference]	Trial ID	Intervention	Comparison	Primary Endpoint	Primary Endpoint Results (month, HR [95%CI])	OS data (month) [a]	OS HR (95% CI)	Note
20	Temozolomide	relapse or progressed glioblastoma multiforme or anaplastic astrocytoma	A Phase 2 study[96]	NA	temozolomide	procarbazine	6-month PFS rate	3.1 vs 2.1 HR not mentioned	7.3 vs 5.7	p = 0.33	Data obtained from label
			China registration study[97]	NA	temozolomide	semustine	6-month PFS rate	78.9% vs 55.9% HR not mentioned	6-month OS% 96.9% vs 97.3%	not reported	
**Colon and rectum**											
21	Raltitrexed	advanced CRC who are not suitable for 5-FU/leucovorin	1694IL/00003[98]	NA	raltitrexed	5-fluorouracil	TTP	4.7 vs 3.6 HR=1.08 (0.89-1.30)	10.3 vs 10.3	1.06 (0.85-1.32)	
			1694IL/0012[99]	NA	raltitrexed	5-fluorouracil+ leucovorin	ORR	18.6% vs 18.1% HR=1.04 (0.65-1.63)	10.9 vs 12.3	1.15 (0.93-1.42)	
			China study[100]	NA	raltitrexed+ oxaliplatin	oxaliplatin+ fluorouracil+ leucovorin	ORR	29.1% vs 17.0% HR not mentioned	not reported	not reported	
22	Cetuximab	EGFR and RAS mutation wild-type mCRC failure to irinotecan-based chemotherapy, in combination with irinotecan	BOND[101]	NA	cetuximab+ irinotecan	cetuximab	ORR	22.9% vs 10.8% HR not mentioned	8.6 vs 6.9	0.91 (0.68-1.21)	
**Thyroid**											

©2022 Zhang Y et al. *JAMA Network Open*.

No	Generic Name (China-only approval)	NMPA approved indication	Pivotal trial [reference]	Trial ID	Intervention	Comparison	Primary Endpoint	Primary Endpoint Results (month, HR [95%CI])	OS data (month) [a]	OS HR (95% CI)	Note
23	Lenvatinib	locally recurrent or metastatic, progressive, radioactive iodine-refractory DTC	SELECT[102]	NCT01321554	lenvatinib	placebo	PFS	18.3 vs 3.6 HR=0.21 (0.14-0.31)	NR vs NR	0.73 (0.50-1.07)	Data obtained from regulatory review document
24	Sorafenib	locally recurrent or metastatic, progressive, radioactive iodine-refractory DTC	DECISION[103]	NCT00984282	sorafenib	placebo	PFS	10.8 vs 5.8 HR=0.59 (0.45-0.76)	NR vs 36.5	0.88 (0.63-1.24)	Data obtained from regulatory review document
**Thyroid**											
25	Everolimus	progressive, well-differentiated, non-functional neuroendocrine tumors of gastrointestinal or lung origin that are unresectable, locally advanced or metastatic	RADIANT-4[104]	NCT01524783	everolimus+ supportive care	placebo+ supportive care	PFS	11.0 vs 3.9 HR=0.48 (0.35-0.67)	NR vs NR	0.64 (0.40-1.05)	
26	Everolimus	unresectable, locally advanced or metastatic,	RADIANT-3[105] [106]	NCT00510068	everolimus	placebo	PFS	11.0 vs 4.6 HR=0.35 (0.27-0.45)	44.0 vs 37.7	0.94 (0.73-1.20)	

©2022 Zhang Y et al. *JAMA Network Open*.

No	Generic Name (China-only approval)	NMPA approved indication	Pivotal trial [reference]	Trial ID	Intervention	Comparison	Primary Endpoint	Primary Endpoint Results (month, HR [95%CI])	OS data (month) [a]	OS HR (95% CI)	Note
		well-differentiated, progressive neuroendocrine tumors of pancreatic origin									
**Prostate**											
27	Apalutamide	nmCRPC, with a high risk of metastasis	SPARTAN[5]	NCT01946204	apalutamide	placebo	MFS	40.5 vs 16.2 HR=0.28 (0.23-0.350	NR vs 39.0	0.70 (0.47-1.04)	
**Ovaries**											
28	Olaparib	maintenance treatment, advanced epithelial ovarian, fallopian tube or primary peritoneal cancer with deleterious or suspected deleterious germline or somatic BRCA-mutated, who are in complete or partial response to first-line platinum-based chemotherapy	SOLO1[107]	NCT01844986	olaparib	placebo	PFS	49.9 vs 13.8 HR=0.31 (0.23-0.41)	3-year OS% 84% vs 80%	0.95 (0.60-1.53)	
**Liver**											
29	Lenvatinib	1st-line, unresectable HCC	REFLECT[108]	NCT01761266	lenvatinib	sorafenib	OS	14.7 vs 10.5 HR=0.82 (0.59-1.14)	14.7 vs 10.5	0.82 (0.59-1.14)	Data obtained from

No	Generic Name (China-only approval)	NMPA approved indication	Pivotal trial [reference]	Trial ID	Intervention	Comparison	Primary Endpoint	Primary Endpoint Results (month, HR [95%CI])	OS data (month) [a]	OS HR (95% CI)	Note
											regulatory review document
**Soft Tissue Sarcoma**											
30	Anlotinib (China-only approval)	2nd-line, acinar soft tissue sarcoma, clear cell sarcoma and other advanced soft tissue sarcoma	ALTER0203	NCT02449343	anlotinib	placebo	PFS	6.9 vs 1.5 HR=0.32 (0.23-0.45)	14.3 vs 12.8	0.96 (0.64-1.41)	Data obtained from regulatory review document
**Lymphoma**											
31	Ibrutinib	2nd-line, MCL	MCL-3001[109]	NCT01646021	ibrutinib	temsirolimus	PFS	14.6 vs 6.2 HR=0.28 (0.23-0.35)	NR vs 21.3	0.76 (0.53-1.09)	
32	Ibrutinib	Waldenström's macroglobulinemia, in combination with rituximab	PCYC-1127-CA[110]	NCT02165397	ibrutinib+ rituximab	placebo+ rituximab	PFS	NR vs 20.3 HR=0.20 (0.11-038)	30-month OS% 94% vs 92%	0.62 (0.17-2.19)	
**Other**											
33	Decitabine	MDS including previously treated and untreated, de novo and secondary MDS of all French-	D-0007[111]	NCT00043381	decitabine	best supportive care	ORR	17% vs 0 HR not mentioned	14.0 vs 14.9	not reported	

©2022 Zhang Y et al. *JAMA Network Open*.

No	Generic Name (China-only approval)	NMPA approved indication	Pivotal trial [reference]	Trial ID	Intervention	Comparison	Primary Endpoint	Primary Endpoint Results (month, HR [95%CI])	OS data (month) [a]	OS HR (95% CI)	Note
		American-British subtypes									
34	Regorafenib	2nd-line, locally advanced, unresectable or metastatic gastrointestinal stromal tumor who have been previously treated with imatinib mesylate and sunitinib malate	GRID[112]	NCT01271712	regorafenib	placebo	PFS	4.8 vs 0.9 HR=0.27 (0.19-0.39)	OS% 78% vs 74%	0.77 (0.42-1.41)	

Abbreviation: *PFS, progression-free survival; NSCLC, non-small cell lung cancer; EGFR, epidermal growth factor receptor; ALK, anaplastic lymphoma kinase; TTP, time-to-progression; IDFS, invasive-disease–free survival; RCC, renal cell carcinoma; RFS, relapse-free survival; NR, not reached; NA, not applicable; CRC, colorectal cancer; mCRC, metastatic colorectal cancer; ORR, objective response rate; DTC, differentiated thyroid cancer; mCRPC, metastatic castration resistant prostate cancer; MFS, metastasis-free survival; HCC, hepatocellular carcinoma; MCL, mantle-cell lymphoma; MDS, myelodysplastic syndromes*

[a] OS benefit measured in months unless otherwise indicated.

©2022 Zhang Y et al. *JAMA Network Open*.

eTable 9. Primary End Point and Time From Enrollment to Approval of 13 Indications With Unknown Overall Survival Data by June 30, 2021

No.	Generic Name (China-only approval)	Indication	Approval Time	Pivotal Trials	Comparator	Primary Endpoint	First Enrolment Time	Duration from enrolment to approval (years)
1	Azacitidine	AML	Apr-2017	CALGB8921[113]	supportive care	response rate	Feb-1994	23.2
2	Dasatinib	adults with chronic, accelerated, or myeloid or lymphoid blast phase Ph+ CML with resistance or intolerance to imatinib	Sep-2011	START-R[114]	imatinib	MCyR%	Feb-2005	6.6
3	Degarelix	advanced prostate cancer	Sep-2018	FE200486 CS21[115]	leuprolide	Cumulative probability of testosterone ≤0.5 ng/mL at any monthly measurement	Feb-2006	12.6
4	Flumatinib (China-only approval)	Philadelphia chromosome-positive chronic phase CML	Nov-2019	FESTnd[7]	imatinib	MMR%	Aug-2014	5.2
5	Fulvestrant	HR-positive advanced breast cancer in postmenopausal women with disease progression following endocrine therapy or relapse after or during anti-estrogen adjuvant therapy	Jun-2010	9238IL/0021[116]	anastrozole	TTP	May-1997	13.1
			Jun-2010	D6997L00004[117]	anastrozole	TTP	Nov-2005	4.6
6	Neratinib	after trastuzumab-based adjuvant therapy in HER2-positive breast cancer	Apr-2020	ExteNET[118 119]	placebo	DFS	Jul-2009	10.8
7	Niraparib	maintenance treatment, platinum-sensitive recurrent epithelial ovarian cancer, fallopian tube cancer or primary peritoneal cancer	Dec-2019	NOVA[8]	placebo	PFS	Aug-2013	6.3
8	Nilotinib	newly diagnosed adult Ph+CML in chronic phase	Jul-2016	CAMN107A2405[120]	imatinib	12month rate of confirmed best cumulative undetectable BCR-ABL1	Jun-2009	7.1
			Jul-2016	ENESTchina[121]	imatinib	MMR%	Apr-2011	5.2

©2022 Zhang Y et al. *JAMA Network Open*.

No.	Generic Name (China-only approval)	Indication	Approval Time	Pivotal Trials	Comparator	Primary Endpoint	First Enrolment Time	Duration from enrolment to approval (years)
			Jul-2016	ENESTnd[122 123]	imatinib	12m MMR%	Sep-2007	8.9
9	Pertuzumab	neoadjuvant treatment, early or locally advanced ERBB2-positive breast cancer, in combination with trastuzumab and chemotherapy	Aug-2019	NeoSphere[124 125]	trastuzumab+ docetaxel pertuzumab+ trastuzumab pertuzumab+ docetaxel	pCR	Dec-2007	11.7
			Aug-2019	PEONY[126]	placebo+ trastuzumab+ docetaxel	pCR	Mar-2016	3.4
10	Palbociclib	initial treatment, HR positive, HER2 negative advanced breast cancer, in combination with letrozole	Jul-2018	PALOMA-2[127]	placebo+ letrzole	PFS	Feb-2013	5.5
11	Chidamide (China-only approval)	advanced, hormone receptor-positive breast cancer, in combination with exemestane	Nov-2019	ACE[128]	placebo	PFS	Jul-2015	4.4
12	Inetetamab (China-only approval)	metastatic breast cancer who has received one or more chemotherapy regimens, in combination with vinorelbine	Jun-2020	A Phase 3 study[129]	vinorelbine	PFS	Jan-2009	11.5
13	Sulfatinib (China-only approval)	advanced extrapancreatic neuroendocrine tumors	Dec-2020	SANET-ep[130]	placebo	PFS	Dec-2015	5.1

Abbreviation: *HR, hazard ratio; ER, estrogen-receptor; HER2, human epidermal growth factor receptor 2; PFS, progression-free survival; pCR, pathologic complete response; CML, chronic myeloid leukemia; MMR, major molecular response; IDFS, invasive disease-free survival.*

©2022 Zhang Y et al. *JAMA Network Open*.

eTable 10. Results of Single-Arm or Dose-Optimization Trials Supporting Approvals by China National Medical Products Administration, 2005-2020

No.	Generic Name (China-only approval)	Indication	Pivotal trial [reference]	Trials ID	Primary endpoint	Result	Complete Response	Partial Response	Note
**Solid tumour**									
1	Icotinib (China-only approval)	2nd-line, locally advanced or metastatic NSCLC	BD-IC-III01-V2[131]	NCT02486354	PFS	5.4 months	0.0%	25.8%	ORR was the secondary endpoint
2	Denosumab	unresectable giant cell tumor of bone	Study 20040215[132]	NCT00396279	ORR	86.0%	Not reported	Not reported	
3	Fluazolepali (China-only approval)	advanced epithelial ovarian, fallopian tube or primary peritoneal cancer with deleterious or suspected deleterious germline or somatic BRCA-mutated	HR-FZPL-Ib-OC	NCT03509636	ORR	69.9%	4.4%	65.5%	Data was obtained from label
4	Crizotinib	ROS1 re- arrangement positive advanced NSCLC	A8081063	CTR20140093	ORR	69.3%	11.0%	58.3%	Data was obtained from label
5	Almonertinib (China-only approval)	2nd-line, locally advanced or metastatic NSCLC with EGFR T790M mutation positive	A phase 2 dose expansion study	NA	ORR	68.9%	Not reported	Not reported	Data was obtained from regulatory review document
6	Ensartinib (China-only approval)	crizotinib-resistant locally advanced or metastatic ALK-positive NSCLC	BTP-42322[133]	NCT03215693	ORR	52.0%	0.0%	52.0%	
7	Tislelizumab (China-only approval)	previously treated locally advanced or metastatic urothelial carcinoma with high PD-L1 expression	BGB-A317-204[134]	NCT04004221; CTR20170071	ORR	24.8%	9.6%	14.4%	
8	Camrelizumab (China-only approval)	previously treated advanced HCC	SHR-1210-II/III-HCC[135]	NCT02989922	ORR	17.6%	0.0%	14.7%	
9	Toripalimab (China-only approval)	previously treated advanced melanoma	POLARIS-01[136]	NCT03013101	ORR	17.3%	0.8%	16.5%	
10	Iodine[131I] Metuximab (China-only approval)	unresectable or relapse HCC	A Phase 2 study	NA	ORR	8.2%	0.0%	8.2%	Data was obtained from label
**Haematological malignancy**									

©2022 Zhang Y et al. *JAMA Network Open*.

No.	Generic Name (China-only approval)	Indication	Pivotal trial [reference]	Trials ID	Primary endpoint	Result	Complete Response	Partial Response	Note
11	Bortezomib	relapsed or refractory MCL	PINNACLE[137]	NCT00063713	TTP	6.2 months	6.4%	25.5%	ORR was also reported
12	Tislelizumab (China-only approval)	3rd-line, relapsed or refractory classical Hodgkin lymphoma	BGB-A317-203[138]	NCT03209973	ORR	87.1%	62.9%	24.3%	
13	Brentuximab Vedotin	relapsed or refractory systemic anaplastic large-cell lymphoma	SG035-0004[139]	NCT00866047	ORR	86.0%	56.9%	29.3%	
14	Zanubrutinib	2nd-line, adult MCL	BGB-3111-206[140]	NCT03206970	ORR	83.7%	68.6%	15.1%	
15	Zanubrutinib	2nd-line, adult CLL/SLL	BGB-3111-205[141]	NCT03206918	ORR	84.0%	3.3%	59.3%	
16	Sintilimab (China-only approval)	3rd-line, relapsed or refractory classical Hodgkin lymphoma	ORIENT-1[142]	NCT03114683	ORR	80.4%	33.7%	46.7%	
17	Orelabrutinib (China-only approval)	2nd-line, adult MCL	ICP-CL-00102	NCT03494179	ORR	77.9%	25.6%	52.3%	Data was obtained from label
18	Camrelizumab (China-only approval)	3rd-line, relapsed or refractory classical Hodgkin lymphoma	SHR-1210-II-204[9]	NCT03155425	ORR	76.0%	21.0%	48.0%	
19	Orelabrutinib (China-only approval)	2nd-line, adult CLL/SLL	ICP-CL-00103	NCT03493217	ORR	73.8%	10.0%	63.8%	Data was obtained from label
20	Bendamustine	2nd-line, adult relapsed indolent non-Hodgkin lymphoma	C18083/3076	NCT01596621	ORR	72.5%	18.6%	53.9%	Data was obtained from regulatory review document
21	Venetoclax	newly diagnosed adult AML patients who are not suitable for strong induction chemotherapy due to comorbidities, or who are 75 years and older, in combination with azacitidine	M14-358[143]	NCT02203773	ORR	63.2%	61.4%	1.8%	

©2022 Zhang Y et al. *JAMA Network Open*.

No.	Generic Name (China-only approval)	Indication	Pivotal trial [reference]	Trials ID	Primary endpoint	Result	Complete Response	Partial Response	Note
22	Pralatrexate	relapsed or refractory PTCL	FOT12-CN-301[144]	NCT03349333	ORR	52.0%	19.7%	32.4%	
23	Brentuximab Vedotin	relapsed or refractory classical Hodgkin lymphoma	C25007[145]	NCT01990534	ORR	50.0%	11.7%	38.3%	
24	Nilotinib	Philadelphia chromosome-positive CML in chronic phase following imatinib resistance and intolerance	CAMN107A2101[146]	NCT00109707	6-month MCyR	48.0%	31.0%	16.0%	
25	Daratumumab	relapsed or refractory multiple myeloma	SIRIUS[147]	NCT01985126	ORR	29.2%	2.8%	26.4%	
26	Chidamide (China-only approval)	2nd-line, relapsed or refractory PTCL	TG0902CDM confirmatory trial	NA	ORR	29.1%	15.2%	13.9%	Data was obtained from label

Abbreviation: *NMPA, National Medical Products Administration; NSCLC, non-small-cell lung cancer; PFS, progression-free survival; ORR, objective response rate; HCC, hepatocellular carcinoma; MMR, major molecular response; MCL, mantle cell lymphoma; CLL, chronic lymphocytic leukemia; SLL, small lymphocytic lymphoma; PTCL, peripheral T-cell lymphoma; CML, chronic myelogenous leukemia; MCyR, major cytogenetic response; NA, not applicable.*

Note: *ORR usually defined as the proportion of patients achieving complete response (CR) or partial response (PR). In some trials, investigators included unconfirmed complete response (CRu) or unconfirmed partial response (PRu) was in the definition of ORR.*

©2022 Zhang Y et al. *JAMA Network Open*.

eTable 11. Published Surrogate Correlation Studies for Cancer Indications Without Documented Overall Survival Benefit

Cancer Site	Setting	Line of therapy	Therapy type	Surrogate measure	Correlation type	Correlation coefficient type	Coefficient	Correlation coefficient (extraction of a root)	Note
**Studies obtained from previous umbrella review[148] (No. Indication=32)**									
Lung[149]	advanced	not specific	targeted	PFS	PFS-HR and OS-HR	R-squared	0.2269	0.48	≥40% crossover
Lung[150]	advanced	1st-line	targeted	PFS	PFS-HR and OS-HR	R-squared	0.002	0.04	molecularly selected patient trials
Lung[151]	advanced	1st-line	targeted	PFS	PFS-OS	Spearman’s r	0.689	0.69	
Lung[152]	advanced	2nd-line	not specific	PFS	PFS-OS	r	0.376	0.38	
Lung[152]	advanced	2nd-line	not specific	PFS	PFS-HR and OS-HR	r	0.415	0.42	
Lung[150]	advanced	not specific	targeted	ORR	ORR-OR and OS-HR	R-squared	0.429	0.65	in trials with molecularly selected patients
Breast[153]	adjuvant setting	adjuvant	not specific	DFS	2-year DFS differences and 5-year OS differences	r	0.62	0.62	base model
Breast[154]	neoadjuvant setting	neoadjuvant	not specific	pCR	logOR(pCR)-logHR(OS)	R-squared	0.09	0.30	
Breast[155]	neoadjuvant setting	neoadjuvant	not specific	pCR	pCR-OS	R-squared	0.24	0.49	trial-level analysis
Breast[156]	advanced	1st-line	targeted	PFS/TTP	PFS/TTP-OS	Spearman’s r	0.81	0.81	
Breast[156]	advanced	1st-line	targeted	PFS/TTP	PFS/TTP-HR and OS-HR	Spearman’s r	0.73	0.73	
Breast[157]	advanced	not specific	anthracyclines, taxanes, or targeted therapies	PFS	PFS-HR and OS-HR	R-squared	0.18	0.42	weighted multivariate regression analysis
Breast[158]	advanced	not specific	not specific	PFS	PFS gain-OS gain	R-squared	0.3	0.55	
Breast[158]	advanced	not specific	not specific	PFS	PFS-HR and OS-HR	R-squared	0.78	0.88	

©2022 Zhang Y et al. *JAMA Network Open*.

**Cancer Site**	**Setting**	**Line of therapy**	**Therapy type**	**Surrogate measure**	**Correlation type**	**Correlation coefficient type**	**Coefficient**	**Correlation coefficient (extraction of a root)**	**Note**
Breast[159]	advanced	not specific	not specific	PFS/TTP	Progression-HR and OS-HR	R-squared	0.3	0.55	
Breast[160]	advanced	not specific	not specific	PFS/TTP	PFS/TTP-OS	r	0.428	0.43	unweighted Spearman
Breast[161]	advanced	2nd-line or 3rd-line	not specific	PFS/TTP	PFS/TTP-OS	Spearman's r	0.7824	0.78	
Breast[161]	advanced	2nd-line or 3rd-line	not specific	PFS/TTP	Progression-HR and OS-HR	Spearman's r	0.5725	0.57	
Breast[157]	advanced	≥2nd-line	anthracyclines, taxanes, or targeted therapies	PFS	PFS-HR and OS-HR	R-squared	0.4	0.63	weighted multivariate regression analysis
Kidney[162]	advanced	1st-line	targeted	PFS	PFS-OS	Spearman's r	0.869	0.87	
Kidney[162]	advanced	1st-line	targeted	PFS	PFS gain-OS gain	Spearman's r	0.36	0.36	
Kidney[163]	advanced	1st-line	targeted	PFS	3-month PFS-9-month OS	Pearson coefficient	0.82	0.82	
Kidney[163]	advanced	1st-line	targeted	PFS	6-month PFS-12-month OS	Pearson coefficient	0.85	0.85	
Kidney[164]	advanced	not specific	cytokine or targeted therapies	PFS/TTP	-ln HR(PFS/TTP) and -ln HR(OS)	weighted Pearson correlation	0.8	0.80	
Kidney[165]	advanced	not specific	targeted	PFS	PFS gain-OS gain	R-squared	0.44	0.66	targeted therapy ordinary linear regression model
Colon and rectum[166]	advanced	not specific	not specific	TTP	TTP HR and OS HR	Spearman ρ	0.8	0.80	
Colon and rectum[167]	advanced	not specific	chemotherapy	ORR	ORR-OR and OS-HR	r	0.42	0.42	
Colon and rectum[168]	advanced	2nd-line	not specific	PFS/TTP	PFS/TTP-OS	R-squared	0.38	0.62	
Colon and rectum[169]	advanced	2nd-line	chemotherapy	ORR	ORR-OS	r	0.58	0.58	
Colon and rectum[170]	advanced	2nd-line	targeted	ORR	ORR-OR and OS-HR	r	0.169	0.17	

©2022 Zhang Y et al. *JAMA Network Open*.

Cancer Site	Setting	Line of therapy	Therapy type	Surrogate measure	Correlation type	Correlation coefficient type	Coefficient	Correlation coefficient (extraction of a root)	Note
Ovaries[158]	advanced	not specific	not specific	PFS	PFS gain-OS gain	R-squared	0.6	0.77	
Ovaries[158]	advanced	not specific	not specific	PFS	PFS-HR and OS-HR	R-squared	0.73	0.85	
Ovaries[171]	advanced	2nd-line or later	not specific	ORR	ORR-OS	R-squared	0.67	0.82	
Urothelial[172]	advanced	not specific	immunotherapy	ORR	ORR-OS	r	-0.12	-0.12	urothelial carcinoma
Prostate[173]	nonmetastatic	1st-line	not specific	MFS	MFS-OS	Kendall's τ	0.91	0.91	weighted linear regression
Prostate[174]	nonmetastatic	1st-line	apalutamide	MFS	MFS-OS	Fleischer method coefficient	0.69	0.69	retrospective analysis of NCT01946204
**Studies obtained from additional search (No. Indication=6, see eBox 2 for search terms)**									
Melanoma[175]	advanced	not specific	not specific	PFS	PFS-OS	R-squared	0.075	0.27	
Melanoma[176]	advanced	not specific	immunotherapy	PFS	PFS-OS	R-squared	0.82	0.91	in sample size
Melanoma[176]	advanced	not specific	immunotherapy	PFS	PFS-HR and OS-HR	R-squared	0.75	0.87	in sample size
Melanoma[176]	advanced	not specific	immunotherapy	PFS	PFS gain-OS gain	R-squared	0.72	0.85	in sample size
Melanoma[176]	advanced	not specific	immunotherapy	ORR	ORR-OS	R-squared	0.25	0.50	in sample size
Soft tissue sarcoma[177]	advanced	2nd-line	not specific	PFS	PFS-OS	r	0.402	0.40	
Brain-glioblastoma[178]	recurrent	not specific	targeted	PFS	PFS-OS	R-squared	0.15	0.39	
Brain-glioblastoma[179]	newly diagnosed or recurrent	not specific	targeted	PFS	6-month PFS%-12-month OS%	r	0.53	0.53	
Liver[180]	unresectable	1st-line and 2nd-line	not specific	PFS	PFS-OS	r	0.84	0.84	PFS with an HR between 0.6-0.7
Liver[181]	unresectable	not specific	immunotherapy	ORR	ORR-OS	Spearman's r	0.35	0.59	
**No relevant study was found by additional search (No. Indication=35)**									
Liver	unresectable	1st-line	targeted	ORR					

©2022 Zhang Y et al. *JAMA Network Open*.

Cancer Site	Setting	Line of therapy	Therapy type	Surrogate measure	Correlation type	Correlation coefficient type	Coefficient	Correlation coefficient (extraction of a root)	Note
Prostate	advanced	1st-line	not specific	testosterone					
Melanoma	advanced	adjuvant	targeted	RFS					
Brain-anaplastic astrocytoma	relapsed	2nd-line	chemotherapy	PFS					
Head and neck-Thyroid	metastatic	1st-line	targeted	PFS					
Neuroendocrine Tumor	advanced	1st-line	targeted	PFS					
Hodgkin lymphoma	relapsed	2nd-line or later	not specific	ORR					
non-Hodgkin lymphoma	relapsed	2nd-line or later	not specific	ORR					
Gastrointestinal stromal tumor	advanced	2nd-line	targeted	PFS					
Waldenström's macroglobulinemia	not specific	1st-line	targeted	PFS					
Myelodysplastic syndromes	not specific	1st-line	chemotherapy	ORR					
leukemia	not specific	not specific	not specific	ORR					
Bone	not specific	not specific	not specific	ORR					
Multiple myeloma	not specific	not specific	not specific	ORR					

Abbreviation: *OS, overall survival; HR, hazard ratio; PFS, progression-free survival; DFS, disease-free survival; pCR, pathologic complete response; ORR, objective response rate; MFS, metastatic-free survival; TTP, time-to-progression; RFS, relapse-free survival.*

©2022 Zhang Y et al. *JAMA Network Open*.

References.

1. Fizazi K, Scher HI, Molina A, et al. Abiraterone acetate for treatment of metastatic castration-resistant prostate cancer: final overall survival analysis of the COU-AA-301 randomised, double-blind, placebo-controlled phase 3 study. *Lancet Oncol* 2012;13(10):983-92. doi: 10.1016/s1470-2045(12)70379-0 [published Online First: 2012/09/22]
2. Yang JC, Wu YL, Schuler M, et al. Afatinib versus cisplatin-based chemotherapy for EGFR mutation-positive lung adenocarcinoma (LUX-Lung 3 and LUX-Lung 6): analysis of overall survival data from two randomised, phase 3 trials. *Lancet Oncol* 2015;16(2):141-51. doi: 10.1016/s1470-2045(14)71173-8 [published Online First: 2015/01/16]
3. Sternberg CN, Hawkins RE, Wagstaff J, et al. A randomised, double-blind phase III study of pazopanib in patients with advanced and/or metastatic renal cell carcinoma: final overall survival results and safety update. *Eur J Cancer* 2013;49(6):1287-96. doi: 10.1016/j.ejca.2012.12.010 [published Online First: 2013/01/17]
4. Shi Y, Zhang L, Liu X, et al. Icotinib versus gefitinib in previously treated advanced non-small-cell lung cancer (ICOGEN): a randomised, double-blind phase 3 non-inferiority trial. *Lancet Oncol* 2013;14(10):953-61. doi: 10.1016/s1470-2045(13)70355-3 [published Online First: 2013/08/21]
5. Smith MR, Saad F, Chowdhury S, et al. Apalutamide Treatment and Metastasis-free Survival in Prostate Cancer. *N Engl J Med* 2018;378(15):1408-18. doi: 10.1056/NEJMoa1715546 [published Online First: 2018/02/09]
6. Long GV, Hauschild A, Santinami M, et al. Adjuvant Dabrafenib plus Trametinib in Stage III BRAF-Mutated Melanoma. *N Engl J Med* 2017;377(19):1813-23. doi: 10.1056/NEJMoa1708539 [published Online First: 2017/09/12]
7. Zhang L, Meng L, Liu B, et al. Flumatinib versus Imatinib for Newly Diagnosed Chronic Phase Chronic Myeloid Leukemia: A Phase III, Randomized, Open-label, Multi-center FESTnd Study. *Clin Cancer Res* 2021;27(1):70-77. doi: 10.1158/1078-0432.Ccr-20-1600 [published Online First: 2020/09/16]
8. Mirza MR, Monk BJ, Herrstedt J, et al. Niraparib Maintenance Therapy in Platinum-Sensitive, Recurrent Ovarian Cancer. *N Engl J Med* 2016;375(22):2154-64. doi: 10.1056/NEJMoa1611310 [published Online First: 2016/10/09]
9. Song Y, Wu J, Chen X, et al. A Single-Arm, Multicenter, Phase II Study of Camrelizumab in Relapsed or Refractory Classical Hodgkin Lymphoma. *Clin Cancer Res* 2019;25(24):7363-69. doi: 10.1158/1078-0432.Ccr-19-1680 [published Online First: 2019/08/20]
10. Soria JC, Felip E, Cobo M, et al. Afatinib versus erlotinib as second-line treatment of patients with advanced squamous cell carcinoma of the lung (LUX-Lung 8): an open-label randomised controlled phase 3 trial. *Lancet Oncol* 2015;16(8):897-907. doi: 10.1016/s1470-2045(15)00006-6 [published Online First: 2015/07/15]
11. Mok T, Camidge DR, Gadgeel SM, et al. Updated overall survival and final progression-free survival data for patients with treatment-naive advanced ALK-positive non-small-cell lung cancer in the ALEX study. *Ann Oncol* 2020;31(8):1056-64. doi: 10.1016/j.annonc.2020.04.478 [published Online First: 2020/05/19]

©2022 Zhang Y et al. *JAMA Network Open*.

12. Horn L, Mansfield AS, Szczęsna A, et al. First-Line Atezolizumab plus Chemotherapy in Extensive-Stage Small-Cell Lung Cancer. *N Engl J Med* 2018;379(23):2220-29. doi: 10.1056/NEJMoa1809064 [published Online First: 2018/10/04]
13. Han B, Li K, Wang Q, et al. Effect of Anlotinib as a Third-Line or Further Treatment on Overall Survival of Patients With Advanced Non-Small Cell Lung Cancer: The ALTER 0303 Phase 3 Randomized Clinical Trial. *JAMA Oncol* 2018;4(11):1569-75. doi: 10.1001/jamaoncol.2018.3039 [published Online First: 2018/08/12]
14. Ramalingam SS, Vansteenkiste J, Planchard D, et al. Overall Survival with Osimertinib in Untreated, EGFR-Mutated Advanced NSCLC. *N Engl J Med* 2020;382(1):41-50. doi: 10.1056/NEJMoa1913662 [published Online First: 2019/11/22]
15. Zhou C, Wu YL, Chen G, et al. BEYOND: A Randomized, Double-Blind, Placebo-Controlled, Multicenter, Phase III Study of First-Line Carboplatin/Paclitaxel Plus Bevacizumab or Placebo in Chinese Patients With Advanced or Recurrent Nonsquamous Non-Small-Cell Lung Cancer. *J Clin Oncol* 2015;33(19):2197-204. doi: 10.1200/jco.2014.59.4424 [published Online First: 2015/05/28]
16. Mok TS, Cheng Y, Zhou X, et al. Updated Overall Survival in a Randomized Study Comparing Dacomitinib with Gefitinib as First-Line Treatment in Patients with Advanced Non-Small-Cell Lung Cancer and EGFR-Activating Mutations. *Drugs* 2021;81(2):257-66. doi: 10.1007/s40265-020-01441-6 [published Online First: 2020/12/18]
17. Gray JE, Villegas A, Daniel D, et al. Three-Year Overall Survival with Durvalumab after Chemoradiotherapy in Stage III NSCLC-Update from PACIFIC. *J Thorac Oncol* 2020;15(2):288-93. doi: 10.1016/j.jtho.2019.10.002 [published Online First: 2019/10/18]
18. Cappuzzo F, Ciuleanu T, Stelmakh L, et al. Erlotinib as maintenance treatment in advanced non-small-cell lung cancer: a multicentre, randomised, placebo-controlled phase 3 study. *Lancet Oncol* 2010;11(6):521-9. doi: 10.1016/s1470-2045(10)70112-1 [published Online First: 2010/05/25]
19. Solomon BJ, Kim DW, Wu YL, et al. Final Overall Survival Analysis From a Study Comparing First-Line Crizotinib Versus Chemotherapy in ALK-Mutation-Positive Non-Small-Cell Lung Cancer. *J Clin Oncol* 2018;36(22):2251-58. doi: 10.1200/jco.2017.77.4794 [published Online First: 2018/05/17]
20. Brahmer J, Reckamp KL, Baas P, et al. Nivolumab versus Docetaxel in Advanced Squamous-Cell Non-Small-Cell Lung Cancer. *N Engl J Med* 2015;373(2):123-35. doi: 10.1056/NEJMoa1504627 [published Online First: 2015/06/02]
21. Gadgeel S, Rodríguez-Abreu D, Speranza G, et al. Updated Analysis From KEYNOTE-189: Pembrolizumab or Placebo Plus Pemetrexed and Platinum for Previously Untreated Metastatic Nonsquamous Non-Small-Cell Lung Cancer. *J Clin Oncol* 2020;38(14):1505-17. doi: 10.1200/jco.19.03136 [published Online First: 2020/03/10]
22. Wu YL, Zhang L, Fan Y, et al. Randomized clinical trial of pembrolizumab vs chemotherapy for previously untreated Chinese patients with PD-L1-positive locally advanced or metastatic non-small-cell lung cancer: KEYNOTE-042 China Study. *Int J Cancer* 2021;148(9):2313-20. doi: 10.1002/ijc.33399 [published Online First: 2020/11/25]

©2022 Zhang Y et al. *JAMA Network Open*.

23. Paz-Ares L, Vicente D, Tafreshi A, et al. A Randomized, Placebo-Controlled Trial of Pembrolizumab Plus Chemotherapy in Patients With Metastatic Squamous NSCLC: Protocol-Specified Final Analysis of KEYNOTE-407. *J Thorac Oncol* 2020;15(10):1657-69. doi: 10.1016/j.jtho.2020.06.015 [published Online First: 2020/07/01]
24. Scagliotti GV, Parikh P, von Pawel J, et al. Phase III study comparing cisplatin plus gemcitabine with cisplatin plus pemetrexed in chemotherapy-naive patients with advanced-stage non-small-cell lung cancer. *J Clin Oncol* 2008;26(21):3543-51. doi: 10.1200/jco.2007.15.0375 [published Online First: 2008/05/29]
25. Ciuleanu T, Brodowicz T, Zielinski C, et al. Maintenance pemetrexed plus best supportive care versus placebo plus best supportive care for non-small-cell lung cancer: a randomised, double-blind, phase 3 study. *Lancet* 2009;374(9699):1432-40. doi: 10.1016/s0140-6736(09)61497-5 [published Online First: 2009/09/22]
26. Fizazi K, Tran N, Fein L, et al. Abiraterone acetate plus prednisone in patients with newly diagnosed high-risk metastatic castration-sensitive prostate cancer (LATITUDE): final overall survival analysis of a randomised, double-blind, phase 3 trial. *Lancet Oncol* 2019;20(5):686-700. doi: 10.1016/s1470-2045(19)30082-8 [published Online First: 2019/04/17]
27. Chi KN, Agarwal N, Bjartell A, et al. Apalutamide for Metastatic, Castration-Sensitive Prostate Cancer. *N Engl J Med* 2019;381(1):13-24. doi: 10.1056/NEJMoa1903307 [published Online First: 2019/06/01]
28. Armstrong AJ, Lin P, Tombal B, et al. Five-year Survival Prediction and Safety Outcomes with Enzalutamide in Men with Chemotherapy-naïve Metastatic Castration-resistant Prostate Cancer from the PREVAIL Trial. *Eur Urol* 2020;78(3):347-57. doi: 10.1016/j.eururo.2020.04.061 [published Online First: 2020/06/13]
29. Sternberg CN, Fizazi K, Saad F, et al. Enzalutamide and Survival in Nonmetastatic, Castration-Resistant Prostate Cancer. *N Engl J Med* 2020;382(23):2197-206. doi: 10.1056/NEJMoa2003892 [published Online First: 2020/05/30]
30. Parker C, Nilsson S, Heinrich D, et al. Alpha emitter radium-223 and survival in metastatic prostate cancer. *N Engl J Med* 2013;369(3):213-23. doi: 10.1056/NEJMoa1213755 [published Online First: 2013/07/19]
31. Hurwitz H, Fehrenbacher L, Novotny W, et al. Bevacizumab plus irinotecan, fluorouracil, and leucovorin for metastatic colorectal cancer. *N Engl J Med* 2004;350(23):2335-42. doi: 10.1056/NEJMoa032691 [published Online First: 2004/06/04]
32. Li J, Qin S, Xu RH, et al. Effect of Fruquintinib vs Placebo on Overall Survival in Patients With Previously Treated Metastatic Colorectal Cancer: The FRESCO Randomized Clinical Trial. *Jama* 2018;319(24):2486-96. doi: 10.1001/jama.2018.7855 [published Online First: 2018/06/28]
33. Mayer RJ, Van Cutsem E, Falcone A, et al. Randomized trial of TAS-102 for refractory metastatic colorectal cancer. *N Engl J Med* 2015;372(20):1909-19. doi: 10.1056/NEJMoa1414325 [published Online First: 2015/05/15]

©2022 Zhang Y et al. *JAMA Network Open*.

34. Li J, Qin S, Xu R, et al. Regorafenib plus best supportive care versus placebo plus best supportive care in Asian patients with previously treated metastatic colorectal cancer (CONCUR): a randomised, double-blind, placebo-controlled, phase 3 trial. *Lancet Oncol* 2015;16(6):619-29. doi: 10.1016/s1470-2045(15)70156-7 [published Online First: 2015/05/20]
35. Facon T, Dimopoulos MA, Dispenzieri A, et al. Final analysis of survival outcomes in the phase 3 FIRST trial of up-front treatment for multiple myeloma. *Blood* 2018;131(3):301-10. doi: 10.1182/blood-2017-07-795047 [published Online First: 2017/11/19]
36. Dimopoulos M, Spencer A, Attal M, et al. Lenalidomide plus dexamethasone for relapsed or refractory multiple myeloma. *N Engl J Med* 2007;357(21):2123-32. doi: 10.1056/NEJMoa070594 [published Online First: 2007/11/23]
37. Richardson PG, Sonneveld P, Schuster M, et al. Extended follow-up of a phase 3 trial in relapsed multiple myeloma: final time-to-event results of the APEX trial. *Blood* 2007;110(10):3557-60. doi: 10.1182/blood-2006-08-036947 [published Online First: 2007/08/11]
38. San Miguel JF, Schlag R, Khuageva NK, et al. Persistent overall survival benefit and no increased risk of second malignancies with bortezomib-melphalan-prednisone versus melphalan-prednisone in patients with previously untreated multiple myeloma. *J Clin Oncol* 2013;31(4):448-55. doi: 10.1200/jco.2012.41.6180 [published Online First: 2012/12/13]
39. San Miguel JF, Schlag R, Khuageva NK, et al. Bortezomib plus melphalan and prednisone for initial treatment of multiple myeloma. *N Engl J Med* 2008;359(9):906-17. doi: 10.1056/NEJMoa0801479 [published Online First: 2008/08/30]
40. Hou J, Jin J, Xu Y, et al. Randomized, double-blind, placebo-controlled phase III study of ixazomib plus lenalidomide-dexamethasone in patients with relapsed/refractory multiple myeloma: China Continuation study. *J Hematol Oncol* 2017;10(1):137. doi: 10.1186/s13045-017-0501-4 [published Online First: 2017/07/08]
41. Cortes J, O'Shaughnessy J, Loesch D, et al. Eribulin monotherapy versus treatment of physician's choice in patients with metastatic breast cancer (EMBRACE): a phase 3 open-label randomised study. *Lancet* 2011;377(9769):914-23. doi: 10.1016/s0140-6736(11)60070-6 [published Online First: 2011/03/08]
42. Swain SM, Miles D, Kim SB, et al. Pertuzumab, trastuzumab, and docetaxel for HER2-positive metastatic breast cancer (CLEOPATRA): end-of-study results from a double-blind, randomised, placebo-controlled, phase 3 study. *Lancet Oncol* 2020;21(4):519-30. doi: 10.1016/s1470-2045(19)30863-0 [published Online First: 2020/03/17]
43. Li J, Qin S, Xu J, et al. Apatinib for chemotherapy-refractory advanced metastatic gastric cancer: results from a randomized, placebo-controlled, parallel-arm, phase II trial. *J Clin Oncol* 2013;31(26):3219-25. doi: 10.1200/jco.2013.48.8585 [published Online First: 2013/08/07]
44. Kang YK, Boku N, Satoh T, et al. Nivolumab in patients with advanced gastric or gastro-oesophageal junction cancer refractory to, or intolerant of, at least two previous chemotherapy regimens (ONO-4538-12, ATTRACTION-2): a randomised, double-blind, placebo-controlled, phase 3 trial. *Lancet* 2017;390(10111):2461-71. doi: 10.1016/s0140-6736(17)31827-5 [published Online First: 2017/10/11]

©2022 Zhang Y et al. *JAMA Network Open*.

45. Burtness B, Harrington KJ, Greil R, et al. Pembrolizumab alone or with chemotherapy versus cetuximab with chemotherapy for recurrent or metastatic squamous cell carcinoma of the head and neck (KEYNOTE-048): a randomised, open-label, phase 3 study. *Lancet* 2019;394(10212):1915-28. doi: 10.1016/s0140-6736(19)32591-7 [published Online First: 2019/11/05]
46. Vermorken JB, Mesia R, Rivera F, et al. Platinum-based chemotherapy plus cetuximab in head and neck cancer. *N Engl J Med* 2008;359(11):1116-27. doi: 10.1056/NEJMoa0802656 [published Online First: 2008/09/12]
47. Fenaux P, Mufti GJ, Hellstrom-Lindberg E, et al. Efficacy of azacitidine compared with that of conventional care regimens in the treatment of higher-risk myelodysplastic syndromes: a randomised, open-label, phase III study. *Lancet Oncol* 2009;10(3):223-32. doi: 10.1016/s1470-2045(09)70003-8 [published Online First: 2009/02/24]
48. Munir T, Brown JR, O'Brien S, et al. Final analysis from RESONATE: Up to six years of follow-up on ibrutinib in patients with previously treated chronic lymphocytic leukemia or small lymphocytic lymphoma. *Am J Hematol* 2019;94(12):1353-63. doi: 10.1002/ajh.25638 [published Online First: 2019/09/13]
49. Kantarjian H, Stein A, Gökbuget N, et al. Blinatumomab versus Chemotherapy for Advanced Acute Lymphoblastic Leukemia. *N Engl J Med* 2017;376(9):836-47. doi: 10.1056/NEJMoa1609783 [published Online First: 2017/03/02]
50. Long GV, Stroyakovskiy D, Gogas H, et al. Dabrafenib and trametinib versus dabrafenib and placebo for Val600 BRAF-mutant melanoma: a multicentre, double-blind, phase 3 randomised controlled trial. *Lancet* 2015;386(9992):444-51. doi: 10.1016/s0140-6736(15)60898-4 [published Online First: 2015/06/04]
51. Chapman PB, Robert C, Larkin J, et al. Vemurafenib in patients with BRAFV600 mutation-positive metastatic melanoma: final overall survival results of the randomized BRIM-3 study. *Ann Oncol* 2017;28(10):2581-87. doi: 10.1093/annonc/mdx339 [published Online First: 2017/09/30]
52. Qin S, Li Q, Gu S, et al. Apatinib as second-line or later therapy in patients with advanced hepatocellular carcinoma (AHELP): a multicentre, double-blind, randomised, placebo-controlled, phase 3 trial. *Lancet Gastroenterol Hepatol* 2021;6(7):559-68. doi: 10.1016/s2468-1253(21)00109-6 [published Online First: 2021/05/11]
53. Finn RS, Qin S, Ikeda M, et al. Atezolizumab plus Bevacizumab in Unresectable Hepatocellular Carcinoma. *N Engl J Med* 2020;382(20):1894-905. doi: 10.1056/NEJMoa1915745 [published Online First: 2020/05/14]
54. Llovet JM, Ricci S, Mazzaferro V, et al. Sorafenib in advanced hepatocellular carcinoma. *N Engl J Med* 2008;359(4):378-90. doi: 10.1056/NEJMoa0708857 [published Online First: 2008/07/25]
55. Huang J, Xu J, Chen Y, et al. Camrelizumab versus investigator's choice of chemotherapy as second-line therapy for advanced or metastatic oesophageal squamous cell carcinoma (ESCORT): a multicentre, randomised, open-label, phase 3 study. *Lancet Oncol* 2020;21(6):832-42. doi: 10.1016/s1470-2045(20)30110-8 [published Online First: 2020/05/18]

©2022 Zhang Y et al. *JAMA Network Open*.

56. Poveda A, Floquet A, Ledermann JA, et al. Olaparib tablets as maintenance therapy in patients with platinum-sensitive relapsed ovarian cancer and a BRCA1/2 mutation (SOLO2/ENGOT-Ov21): a final analysis of a double-blind, randomised, placebo-controlled, phase 3 trial. *Lancet Oncol* 2021;22(5):620-31. doi: 10.1016/s1470-2045(21)00073-5 [published Online First: 2021/03/22]
57. González-Martín A, Pothuri B, Vergote I, et al. Niraparib in Patients with Newly Diagnosed Advanced Ovarian Cancer. *N Engl J Med* 2019;381(25):2391-402. doi: 10.1056/NEJMoa1910962 [published Online First: 2019/09/29]
58. Robak T, Jin J, Pylypenko H, et al. Frontline bortezomib, rituximab, cyclophosphamide, doxorubicin, and prednisone (VR-CAP) versus rituximab, cyclophosphamide, doxorubicin, vincristine, and prednisone (R-CHOP) in transplantation-ineligible patients with newly diagnosed mantle cell lymphoma: final overall survival results of a randomised, open-label, phase 3 study. *Lancet Oncol* 2018;19(11):1449-58. doi: 10.1016/s1470-2045(18)30685-5 [published Online First: 2018/10/24]
59. Stupp R, Mason WP, van den Bent MJ, et al. Radiotherapy plus concomitant and adjuvant temozolomide for glioblastoma. *N Engl J Med* 2005;352(10):987-96. doi: 10.1056/NEJMoa043330 [published Online First: 2005/03/11]
60. Vogelzang NJ, Rusthoven JJ, Symanowski J, et al. Phase III study of pemetrexed in combination with cisplatin versus cisplatin alone in patients with malignant pleural mesothelioma. *J Clin Oncol* 2003;21(14):2636-44. doi: 10.1200/jco.2003.11.136 [published Online First: 2003/07/16]
61. Faivre S, Niccoli P, Castellano D, et al. Sunitinib in pancreatic neuroendocrine tumors: updated progression-free survival and final overall survival from a phase III randomized study. *Ann Oncol* 2017;28(2):339-43. doi: 10.1093/annonc/mdw561 [published Online First: 2016/11/12]
62. Raymond E, Dahan L, Raoul JL, et al. Sunitinib malate for the treatment of pancreatic neuroendocrine tumors. *N Engl J Med* 2011;364(6):501-13. doi: 10.1056/NEJMoa1003825 [published Online First: 2011/02/11]
63. Demetri GD, van Oosterom AT, Garrett CR, et al. Efficacy and safety of sunitinib in patients with advanced gastrointestinal stromal tumour after failure of imatinib: a randomised controlled trial. *Lancet* 2006;368(9544):1329-38. doi: 10.1016/s0140-6736(06)69446-4 [published Online First: 2006/10/19]
64. Zhou C, Chen G, Huang Y, et al. Camrelizumab plus carboplatin and pemetrexed versus chemotherapy alone in chemotherapy-naive patients with advanced non-squamous non-small-cell lung cancer (CameL): a randomised, open-label, multicentre, phase 3 trial. *Lancet Respir Med* 2021;9(3):305-14. doi: 10.1016/s2213-2600(20)30365-9 [published Online First: 2020/12/22]
65. Soria JC, Tan DSW, Chiari R, et al. First-line ceritinib versus platinum-based chemotherapy in advanced ALK-rearranged non-small-cell lung cancer (ASCEND-4): a randomised, open-label, phase 3 study. *Lancet* 2017;389(10072):917-29. doi: 10.1016/s0140-6736(17)30123-x [published Online First: 2017/01/28]
66. Shaw AT, Kim TM, Crinò L, et al. Ceritinib versus chemotherapy in patients with ALK-rearranged non-small-cell lung cancer previously given chemotherapy and crizotinib (ASCEND-5): a randomised, controlled, open-label, phase 3 trial. *Lancet Oncol* 2017;18(7):874-86. doi: 10.1016/s1470-2045(17)30339-x [published Online First: 2017/06/13]

©2022 Zhang Y et al. *JAMA Network Open*.

67. Wu YL, Zhou C, Liam CK, et al. First-line erlotinib versus gemcitabine/cisplatin in patients with advanced EGFR mutation-positive non-small-cell lung cancer: analyses from the phase III, randomized, open-label, ENSURE study. *Ann Oncol* 2015;26(9):1883-89. doi: 10.1093/annonc/mdv270 [published Online First: 2015/06/25]
68. Rosell R, Carcereny E, Gervais R, et al. Erlotinib versus standard chemotherapy as first-line treatment for European patients with advanced EGFR mutation-positive non-small-cell lung cancer (EURTAC): a multicentre, open-label, randomised phase 3 trial. *Lancet Oncol* 2012;13(3):239-46. doi: 10.1016/s1470-2045(11)70393-x [published Online First: 2012/01/31]
69. Zhou C, Wu YL, Chen G, et al. Erlotinib versus chemotherapy as first-line treatment for patients with advanced EGFR mutation-positive non-small-cell lung cancer (OPTIMAL, CTONG-0802): a multicentre, open-label, randomised, phase 3 study. *Lancet Oncol* 2011;12(8):735-42. doi: 10.1016/s1470-2045(11)70184-x [published Online First: 2011/07/26]
70. Zhou C, Wu YL, Chen G, et al. Final overall survival results from a randomised, phase III study of erlotinib versus chemotherapy as first-line treatment of EGFR mutation-positive advanced non-small-cell lung cancer (OPTIMAL, CTONG-0802). *Ann Oncol* 2015;26(9):1877-83. doi: 10.1093/annonc/mdv276 [published Online First: 2015/07/05]
71. Mok TS, Wu YL, Ahn MJ, et al. Osimertinib or Platinum-Pemetrexed in EGFR T790M-Positive Lung Cancer. *N Engl J Med* 2017;376(7):629-40. doi: 10.1056/NEJMoa1612674 [published Online First: 2016/12/14]
72. Papadimitrakopoulou VA, Mok TS, Han JY, et al. Osimertinib versus platinum-pemetrexed for patients with EGFR T790M advanced NSCLC and progression on a prior EGFR-tyrosine kinase inhibitor: AURA3 overall survival analysis. *Ann Oncol* 2020;31(11):1536-44. doi: 10.1016/j.annonc.2020.08.2100 [published Online First: 2020/08/31]
73. Sun Y, Wu YL, Zhou CC, et al. Second-line pemetrexed versus docetaxel in Chinese patients with locally advanced or metastatic non-small cell lung cancer: a randomized, open-label study. *Lung Cancer* 2013;79(2):143-50. doi: 10.1016/j.lungcan.2012.10.015 [published Online First: 2012/11/28]
74. Hanna N, Shepherd FA, Fossella FV, et al. Randomized phase III trial of pemetrexed versus docetaxel in patients with non-small-cell lung cancer previously treated with chemotherapy. *J Clin Oncol* 2004;22(9):1589-97. doi: 10.1200/jco.2004.08.163 [published Online First: 2004/05/01]
75. Johnston S, Martin M, Di Leo A, et al. MONARCH 3 final PFS: a randomized study of abemaciclib as initial therapy for advanced breast cancer. *NPJ Breast Cancer* 2019;5:5. doi: 10.1038/s41523-018-0097-z [published Online First: 2019/01/25]
76. Goetz MP, Toi M, Campone M, et al. MONARCH 3: Abemaciclib As Initial Therapy for Advanced Breast Cancer. *J Clin Oncol* 2017;35(32):3638-46. doi: 10.1200/jco.2017.75.6155 [published Online First: 2017/10/03]
77. Zhang QY, Sun T, Yin YM, et al. MONARCH plus: abemaciclib plus endocrine therapy in women with HR+/HER2- advanced breast cancer: the multinational randomized phase III study. *Ther Adv Med Oncol* 2020;12:1758835920963925. doi: 10.1177/1758835920963925 [published Online First: 2020/11/06]

©2022 Zhang Y et al. *JAMA Network Open*.

78. Cameron D, Casey M, Press M, et al. A phase III randomized comparison of lapatinib plus capecitabine versus capecitabine alone in women with advanced breast cancer that has progressed on trastuzumab: updated efficacy and biomarker analyses. *Breast Cancer Res Treat* 2008;112(3):533-43. doi: 10.1007/s10549-007-9885-0 [published Online First: 2008/01/12]
79. Cameron D, Casey M, Oliva C, et al. Lapatinib plus capecitabine in women with HER-2-positive advanced breast cancer: final survival analysis of a phase III randomized trial. *Oncologist* 2010;15(9):924-34. doi: 10.1634/theoncologist.2009-0181 [published Online First: 2010/08/26]
80. von Minckwitz G, Procter M, de Azambuja E, et al. Adjuvant Pertuzumab and Trastuzumab in Early HER2-Positive Breast Cancer. *N Engl J Med* 2017;377(2):122-31. doi: 10.1056/NEJMoa1703643 [published Online First: 2017/06/06]
81. Piccart M, Procter M, Fumagalli D, et al. Adjuvant Pertuzumab and Trastuzumab in Early HER2-Positive Breast Cancer in the APHINITY Trial: 6 Years' Follow-Up. *J Clin Oncol* 2021;39(13):1448-57. doi: 10.1200/jco.20.01204 [published Online First: 2021/02/05]
82. von Minckwitz G, Huang CS, Mano MS, et al. Trastuzumab Emtansine for Residual Invasive HER2-Positive Breast Cancer. *N Engl J Med* 2019;380(7):617-28. doi: 10.1056/NEJMoa1814017 [published Online First: 2018/12/06]
83. Qin S, Bi F, Jin J, et al. Axitinib versus sorafenib as a second-line therapy in Asian patients with metastatic renal cell carcinoma: results from a randomized registrational study. *Onco Targets Ther* 2015;8:1363-73. doi: 10.2147/ott.S83302 [published Online First: 2015/06/20]
84. Hutson TE, Al-Shukri S, Stus VP, et al. Axitinib Versus Sorafenib in First-Line Metastatic Renal Cell Carcinoma: Overall Survival From a Randomized Phase III Trial. *Clin Genitourin Cancer* 2017;15(1):72-76. doi: 10.1016/j.clgc.2016.05.008 [published Online First: 2016/08/09]
85. Rini BI, Escudier B, Tomczak P, et al. Comparative effectiveness of axitinib versus sorafenib in advanced renal cell carcinoma (AXIS): a randomised phase 3 trial. *Lancet* 2011;378(9807):1931-9. doi: 10.1016/s0140-6736(11)61613-9 [published Online First: 2011/11/08]
86. Motzer RJ, Escudier B, Tomczak P, et al. Axitinib versus sorafenib as second-line treatment for advanced renal cell carcinoma: overall survival analysis and updated results from a randomised phase 3 trial. *Lancet Oncol* 2013;14(6):552-62. doi: 10.1016/s1470-2045(13)70093-7 [published Online First: 2013/04/20]
87. Motzer RJ, Hutson TE, Tomczak P, et al. Sunitinib versus interferon alfa in metastatic renal-cell carcinoma. *N Engl J Med* 2007;356(2):115-24. doi: 10.1056/NEJMoa065044 [published Online First: 2007/01/12]
88. Escudier B, Eisen T, Stadler WM, et al. Sorafenib in advanced clear-cell renal-cell carcinoma. *N Engl J Med* 2007;356(2):125-34. doi: 10.1056/NEJMoa060655 [published Online First: 2007/01/12]
89. Motzer RJ, Escudier B, Oudard S, et al. Efficacy of everolimus in advanced renal cell carcinoma: a double-blind, randomised, placebo-controlled phase III trial. *Lancet* 2008;372(9637):449-56. doi: 10.1016/s0140-6736(08)61039-9 [published Online First: 2008/07/26]
90. Motzer RJ, Escudier B, Oudard S, et al. Phase 3 trial of everolimus for metastatic renal cell carcinoma : final results and analysis of prognostic factors. *Cancer* 2010;116(18):4256-65. doi: 10.1002/cncr.25219 [published Online First: 2010/06/16]

©2022 Zhang Y et al. *JAMA Network Open*.

91. Dummer R, Hauschild A, Santinami M, et al. Five-Year Analysis of Adjuvant Dabrafenib plus Trametinib in Stage III Melanoma. *N Engl J Med* 2020;383(12):1139-48. doi: 10.1056/NEJMoa2005493 [published Online First: 2020/09/03]
92. Ribas A, Puzanov I, Dummer R, et al. Pembrolizumab versus investigator-choice chemotherapy for ipilimumab-refractory melanoma (KEYNOTE-002): a randomised, controlled, phase 2 trial. *Lancet Oncol* 2015;16(8):908-18. doi: 10.1016/s1470-2045(15)00083-2 [published Online First: 2015/06/28]
93. Hamid O, Puzanov I, Dummer R, et al. Final analysis of a randomised trial comparing pembrolizumab versus investigator-choice chemotherapy for ipilimumab-refractory advanced melanoma. *Eur J Cancer* 2017;86:37-45. doi: 10.1016/j.ejca.2017.07.022 [published Online First: 2017/09/30]
94. Ellingson BM, Kim HJ, Woodworth DC, et al. Recurrent glioblastoma treated with bevacizumab: contrast-enhanced T1-weighted subtraction maps improve tumor delineation and aid prediction of survival in a multicenter clinical trial. *Radiology* 2014;271(1):200-10. doi: 10.1148/radiol.13131305 [published Online First: 2014/01/31]
95. Wick W, Gorlia T, Bendszus M, et al. Lomustine and Bevacizumab in Progressive Glioblastoma. *N Engl J Med* 2017;377(20):1954-63. doi: 10.1056/NEJMoa1707358 [published Online First: 2017/11/16]
96. Yung WK, Albright RE, Olson J, et al. A phase II study of temozolomide vs. procarbazine in patients with glioblastoma multiforme at first relapse. *Br J Cancer* 2000;83(5):588-93. doi: 10.1054/bjoc.2000.1316 [published Online First: 2000/08/17]
97. Sun J, Yang XJ, Yang SY. [Multicenter randomized controlled study of temozolomide versus semustine in the treatment of recurrent malignant glioma]. *Zhonghua Yi Xue Za Zhi* 2013;93(3):165-8. [published Online First: 2013/04/11]
98. Cunningham D, Zalcberg JR, Rath U, et al. Final results of a randomised trial comparing 'Tomudex' (raltitrexed) with 5-fluorouracil plus leucovorin in advanced colorectal cancer. "Tomudex" Colorectal Cancer Study Group. *Ann Oncol* 1996;7(9):961-5. doi: 10.1093/oxfordjournals.annonc.a010800 [published Online First: 1996/11/01]
99. Cocconi G, Cunningham D, Van Cutsem E, et al. Open, randomized, multicenter trial of raltitrexed versus fluorouracil plus high-dose leucovorin in patients with advanced colorectal cancer. Tomudex Colorectal Cancer Study Group. *J Clin Oncol* 1998;16(9):2943-52. doi: 10.1200/jco.1998.16.9.2943 [published Online First: 1998/09/17]
100. Jia-lei W, Jin L, Shu-kui Q, et al. Randomized multicenter phase III trial of oxaliplatin plus raltitrexed versus oxaliplatin plus fluorouracil and leucovorin for advanced colorectal cancer. *Chinese Clinical Oncology* 2012;17(01):6-11.
101. Cunningham D, Humblet Y, Siena S, et al. Cetuximab monotherapy and cetuximab plus irinotecan in irinotecan-refractory metastatic colorectal cancer. *N Engl J Med* 2004;351(4):337-45. doi: 10.1056/NEJMoa033025 [published Online First: 2004/07/23]
102. Schlumberger M, Tahara M, Wirth LJ, et al. Lenvatinib versus placebo in radioiodine-refractory thyroid cancer. *N Engl J Med* 2015;372(7):621-30. doi: 10.1056/NEJMoa1406470 [published Online First: 2015/02/12]
103. Brose MS, Nutting CM, Jarzab B, et al. Sorafenib in radioactive iodine-refractory, locally advanced or metastatic differentiated thyroid cancer: a randomised, double-blind, phase 3 trial. *Lancet* 2014;384(9940):319-28. doi: 10.1016/s0140-6736(14)60421-9 [published Online First: 2014/04/29]

©2022 Zhang Y et al. *JAMA Network Open*.

104. Yao JC, Fazio N, Singh S, et al. Everolimus for the treatment of advanced, non-functional neuroendocrine tumours of the lung or gastrointestinal tract (RADIANT-4): a randomised, placebo-controlled, phase 3 study. *Lancet* 2016;387(10022):968-77. doi: 10.1016/s0140-6736(15)00817-x [published Online First: 2015/12/26]
105. Yao JC, Shah MH, Ito T, et al. Everolimus for advanced pancreatic neuroendocrine tumors. *N Engl J Med* 2011;364(6):514-23. doi: 10.1056/NEJMoa1009290 [published Online First: 2011/02/11]
106. Yao JC, Pavel M, Lombard-Bohas C, et al. Everolimus for the Treatment of Advanced Pancreatic Neuroendocrine Tumors: Overall Survival and Circulating Biomarkers From the Randomized, Phase III RADIANT-3 Study. *J Clin Oncol* 2016;34(32):3906-13. doi: 10.1200/jco.2016.68.0702 [published Online First: 2016/09/14]
107. Moore K, Colombo N, Scambia G, et al. Maintenance Olaparib in Patients with Newly Diagnosed Advanced Ovarian Cancer. *N Engl J Med* 2018;379(26):2495-505. doi: 10.1056/NEJMoa1810858 [published Online First: 2018/10/23]
108. Kudo M, Finn RS, Qin S, et al. Lenvatinib versus sorafenib in first-line treatment of patients with unresectable hepatocellular carcinoma: a randomised phase 3 non-inferiority trial. *Lancet* 2018;391(10126):1163-73. doi: 10.1016/s0140-6736(18)30207-1 [published Online First: 2018/02/13]
109. Dreyling M, Jurczak W, Jerkeman M, et al. Ibrutinib versus temsirolimus in patients with relapsed or refractory mantle-cell lymphoma: an international, randomised, open-label, phase 3 study. *Lancet* 2016;387(10020):770-8. doi: 10.1016/s0140-6736(15)00667-4 [published Online First: 2015/12/18]
110. Dimopoulos MA, Tedeschi A, Trotman J, et al. Phase 3 Trial of Ibrutinib plus Rituximab in Waldenström's Macroglobulinemia. *N Engl J Med* 2018;378(25):2399-410. doi: 10.1056/NEJMoa1802917 [published Online First: 2018/06/02]
111. Kantarjian H, Issa JP, Rosenfeld CS, et al. Decitabine improves patient outcomes in myelodysplastic syndromes: results of a phase III randomized study. *Cancer* 2006;106(8):1794-803. doi: 10.1002/cncr.21792 [published Online First: 2006/03/15]
112. Demetri GD, Reichardt P, Kang YK, et al. Efficacy and safety of regorafenib for advanced gastrointestinal stromal tumours after failure of imatinib and sunitinib (GRID): an international, multicentre, randomised, placebo-controlled, phase 3 trial. *Lancet* 2013;381(9863):295-302. doi: 10.1016/s0140-6736(12)61857-1 [published Online First: 2012/11/28]
113. Silverman LR, Demakos EP, Peterson BL, et al. Randomized controlled trial of azacitidine in patients with the myelodysplastic syndrome: a study of the cancer and leukemia group B. *J Clin Oncol* 2002;20(10):2429-40. doi: 10.1200/jco.2002.04.117 [published Online First: 2002/05/16]
114. Kantarjian H, Pasquini R, Lévy V, et al. Dasatinib or high-dose imatinib for chronic-phase chronic myeloid leukemia resistant to imatinib at a dose of 400 to 600 milligrams daily: two-year follow-up of a randomized phase 2 study (START-R). *Cancer* 2009;115(18):4136-47. doi: 10.1002/cncr.24504 [published Online First: 2009/06/19]
115. Klotz L, Boccon-Gibod L, Shore ND, et al. The efficacy and safety of degarelix: a 12-month, comparative, randomized, open-label, parallel-group phase III study in patients with prostate cancer. *BJU Int* 2008;102(11):1531-8. doi: 10.1111/j.1464-410X.2008.08183.x [published Online First: 2008/11/28]

©2022 Zhang Y et al. *JAMA Network Open*.

116. Osborne CK, Pippen J, Jones SE, et al. Double-blind, randomized trial comparing the efficacy and tolerability of fulvestrant versus anastrozole in postmenopausal women with advanced breast cancer progressing on prior endocrine therapy: results of a North American trial. *J Clin Oncol* 2002;20(16):3386-95. doi: 10.1200/jco.2002.10.058 [published Online First: 2002/08/15]
117. Xu B, Jiang Z, Shao Z, et al. Fulvestrant 250 mg versus anastrozole for Chinese patients with advanced breast cancer: results of a multicentre, double-blind, randomised phase III trial. *Cancer Chemother Pharmacol* 2011;67(1):223-30. doi: 10.1007/s00280-010-1483-x [published Online First: 2010/10/13]
118. Martin M, Holmes FA, Ejlertsen B, et al. Neratinib after trastuzumab-based adjuvant therapy in HER2-positive breast cancer (ExteNET): 5-year analysis of a randomised, double-blind, placebo-controlled, phase 3 trial. *Lancet Oncol* 2017;18(12):1688-700. doi: 10.1016/s1470-2045(17)30717-9 [published Online First: 2017/11/18]
119. Iwata H, Masuda N, Kim SB, et al. Neratinib after trastuzumab-based adjuvant therapy in patients from Asia with early stage HER2-positive breast cancer. *Future Oncol* 2019;15(21):2489-501. doi: 10.2217/fon-2019-0143 [published Online First: 2019/05/30]
120. Hughes TP, Lipton JH, Spector N, et al. Deep molecular responses achieved in patients with CML-CP who are switched to nilotinib after long-term imatinib. *Blood* 2014;124(5):729-36. doi: 10.1182/blood-2013-12-544015 [published Online First: 2014/06/21]
121. Wang J, Shen ZX, Saglio G, et al. Phase 3 study of nilotinib vs imatinib in Chinese patients with newly diagnosed chronic myeloid leukemia in chronic phase: ENESTchina. *Blood* 2015;125(18):2771-8. doi: 10.1182/blood-2014-09-601674 [published Online First: 2015/03/15]
122. Saglio G, Kim DW, Issaragrisil S, et al. Nilotinib versus imatinib for newly diagnosed chronic myeloid leukemia. *N Engl J Med* 2010;362(24):2251-9. doi: 10.1056/NEJMoa0912614 [published Online First: 2010/06/08]
123. Kantarjian HM, Hochhaus A, Saglio G, et al. Nilotinib versus imatinib for the treatment of patients with newly diagnosed chronic phase, Philadelphia chromosome-positive, chronic myeloid leukaemia: 24-month minimum follow-up of the phase 3 randomised ENESTnd trial. *Lancet Oncol* 2011;12(9):841-51. doi: 10.1016/s1470-2045(11)70201-7 [published Online First: 2011/08/23]
124. Gianni L, Pienkowski T, Im YH, et al. Efficacy and safety of neoadjuvant pertuzumab and trastuzumab in women with locally advanced, inflammatory, or early HER2-positive breast cancer (NeoSphere): a randomised multicentre, open-label, phase 2 trial. *Lancet Oncol* 2012;13(1):25-32. doi: 10.1016/s1470-2045(11)70336-9 [published Online First: 2011/12/14]
125. Gianni L, Pienkowski T, Im YH, et al. 5-year analysis of neoadjuvant pertuzumab and trastuzumab in patients with locally advanced, inflammatory, or early-stage HER2-positive breast cancer (NeoSphere): a multicentre, open-label, phase 2 randomised trial. *Lancet Oncol* 2016;17(6):791-800. doi: 10.1016/s1470-2045(16)00163-7 [published Online First: 2016/05/18]
126. Shao Z, Pang D, Yang H, et al. Efficacy, Safety, and Tolerability of Pertuzumab, Trastuzumab, and Docetaxel for Patients With Early or Locally Advanced ERBB2-Positive Breast Cancer in Asia: The PEONY Phase 3 Randomized Clinical Trial. *JAMA Oncol* 2020;6(3):e193692. doi: 10.1001/jamaoncol.2019.3692 [published Online First: 2019/10/28]

©2022 Zhang Y et al. *JAMA Network Open*.

127. Finn RS, Martin M, Rugo HS, et al. Palbociclib and Letrozole in Advanced Breast Cancer. *N Engl J Med* 2016;375(20):1925-36. doi: 10.1056/NEJMoa1607303 [published Online First: 2016/12/14]
128. Jiang Z, Li W, Hu X, et al. Tucidinostat plus exemestane for postmenopausal patients with advanced, hormone receptor-positive breast cancer (ACE): a randomised, double-blind, placebo-controlled, phase 3 trial. *Lancet Oncol* 2019;20(6):806-15. doi: 10.1016/s1470-2045(19)30164-0 [published Online First: 2019/05/01]
129. Bian L, Xu BH, Di LJ, et al. [Phase III randomized controlled, multicenter, prospective study of recombinant anti-HER2 humanized monoclonal antibody (Cipterbin) combined with vinorelbine in patients with HER2 positive metastatic breast cancer: the HOPES Study]. *Zhonghua Yi Xue Za Zhi* 2020;100(30):2351-57. doi: 10.3760/cma.j.cn112137-20200116-00105 [published Online First: 2020/08/15]
130. Xu J, Shen L, Zhou Z, et al. Surufatinib in advanced extrapancreatic neuroendocrine tumours (SANET-ep): a randomised, double-blind, placebo-controlled, phase 3 study. *Lancet Oncol* 2020;21(11):1500-12. doi: 10.1016/s1470-2045(20)30496-4 [published Online First: 2020/09/24]
131. Hu X, Zhang L, Shi Y, et al. The Efficacy and Safety of Icotinib in Patients with Advanced Non-Small Cell Lung Cancer Previously Treated with Chemotherapy: A Single-Arm, Multi-Center, Prospective Study. *PLoS One* 2015;10(11):e0142500. doi: 10.1371/journal.pone.0142500 [published Online First: 2015/11/26]
132. Thomas D, Henshaw R, Skubitz K, et al. Denosumab in patients with giant-cell tumour of bone: an open-label, phase 2 study. *Lancet Oncol* 2010;11(3):275-80. doi: 10.1016/s1470-2045(10)70010-3 [published Online First: 2010/02/13]
133. Yang Y, Zhou J, Zhou J, et al. Efficacy, safety, and biomarker analysis of ensartinib in crizotinib-resistant, ALK-positive non-small-cell lung cancer: a multicentre, phase 2 trial. *Lancet Respir Med* 2020;8(1):45-53. doi: 10.1016/s2213-2600(19)30252-8 [published Online First: 2019/10/20]
134. Ye D, Liu J, Zhou A, et al. Tislelizumab in Asian patients with previously treated locally advanced or metastatic urothelial carcinoma. *Cancer Sci* 2021;112(1):305-13. doi: 10.1111/cas.14681 [published Online First: 2020/10/14]
135. Qin S, Ren Z, Meng Z, et al. Camrelizumab in patients with previously treated advanced hepatocellular carcinoma: a multicentre, open-label, parallel-group, randomised, phase 2 trial. *Lancet Oncol* 2020;21(4):571-80. doi: 10.1016/s1470-2045(20)30011-5 [published Online First: 2020/03/01]
136. Tang B, Chi Z, Chen Y, et al. Safety, Efficacy, and Biomarker Analysis of Toripalimab in Previously Treated Advanced Melanoma: Results of the POLARIS-01 Multicenter Phase II Trial. *Clin Cancer Res* 2020;26(16):4250-59. doi: 10.1158/1078-0432.Ccr-19-3922 [published Online First: 2020/04/24]
137. Fisher RI, Bernstein SH, Kahl BS, et al. Multicenter phase II study of bortezomib in patients with relapsed or refractory mantle cell lymphoma. *J Clin Oncol* 2006;24(30):4867-74. doi: 10.1200/jco.2006.07.9665 [published Online First: 2006/09/27]
138. Song Y, Gao Q, Zhang H, et al. Treatment of relapsed or refractory classical Hodgkin lymphoma with the anti-PD-1, tislelizumab: results of a phase 2, single-arm, multicenter study. *Leukemia* 2020;34(2):533-42. doi: 10.1038/s41375-019-0545-2 [published Online First: 2019/09/15]

©2022 Zhang Y et al. *JAMA Network Open*.

139. Pro B, Advani R, Brice P, et al. Brentuximab vedotin (SGN-35) in patients with relapsed or refractory systemic anaplastic large-cell lymphoma: results of a phase II study. *J Clin Oncol* 2012;30(18):2190-6. doi: 10.1200/jco.2011.38.0402 [published Online First: 2012/05/23]
140. Song Y, Zhou K, Zou D, et al. Treatment of Patients with Relapsed or Refractory Mantle-Cell Lymphoma with Zanubrutinib, a Selective Inhibitor of Bruton's Tyrosine Kinase. *Clin Cancer Res* 2020;26(16):4216-24. doi: 10.1158/1078-0432.Ccr-19-3703 [published Online First: 2020/05/29]
141. Xu W, Yang S, Zhou K, et al. Treatment of relapsed/refractory chronic lymphocytic leukemia/small lymphocytic lymphoma with the BTK inhibitor zanubrutinib: phase 2, single-arm, multicenter study. *J Hematol Oncol* 2020;13(1):48. doi: 10.1186/s13045-020-00884-4 [published Online First: 2020/05/13]
142. Shi Y, Su H, Song Y, et al. Safety and activity of sintilimab in patients with relapsed or refractory classical Hodgkin lymphoma (ORIENT-1): a multicentre, single-arm, phase 2 trial. *Lancet Haematol* 2019;6(1):e12-e19. doi: 10.1016/s2352-3026(18)30192-3 [published Online First: 2019/01/08]
143. DiNardo CD, Pratz KW, Letai A, et al. Safety and preliminary efficacy of venetoclax with decitabine or azacitidine in elderly patients with previously untreated acute myeloid leukaemia: a non-randomised, open-label, phase 1b study. *Lancet Oncol* 2018;19(2):216-28. doi: 10.1016/s1470-2045(18)30010-x [published Online First: 2018/01/18]
144. Hong X, Song Y, Huang H, et al. Pralatrexate in Chinese Patients with Relapsed or Refractory Peripheral T-cell Lymphoma: A Single-arm, Multicenter Study. *Target Oncol* 2019;14(2):149-58. doi: 10.1007/s11523-019-00630-y [published Online First: 2019/03/25]
145. Walewski J, Hellmann A, Siritanaratkul N, et al. Prospective study of brentuximab vedotin in relapsed/refractory Hodgkin lymphoma patients who are not suitable for stem cell transplant or multi-agent chemotherapy. *Br J Haematol* 2018;183(3):400-10. doi: 10.1111/bjh.15539 [published Online First: 2018/09/01]
146. Kantarjian HM, Giles F, Gattermann N, et al. Nilotinib (formerly AMN107), a highly selective BCR-ABL tyrosine kinase inhibitor, is effective in patients with Philadelphia chromosome-positive chronic myelogenous leukemia in chronic phase following imatinib resistance and intolerance. *Blood* 2007;110(10):3540-6. doi: 10.1182/blood-2007-03-080689 [published Online First: 2007/08/24]
147. Lonial S, Weiss BM, Usmani SZ, et al. Daratumumab monotherapy in patients with treatment-refractory multiple myeloma (SIRIUS): an open-label, randomised, phase 2 trial. *Lancet* 2016;387(10027):1551-60. doi: 10.1016/s0140-6736(15)01120-4 [published Online First: 2016/01/19]
148. Haslam A, Hey SP, Gill J, et al. A systematic review of trial-level meta-analyses measuring the strength of association between surrogate end-points and overall survival in oncology. *Eur J Cancer* 2019;106:196-211. doi: 10.1016/j.ejca.2018.11.012 [published Online First: 2018/12/12]
149. Hotta K, Suzuki E, Di Maio M, et al. Progression-free survival and overall survival in phase III trials of molecular-targeted agents in advanced non-small-cell lung cancer. *Lung Cancer* 2013;79(1):20-6. doi: 10.1016/j.lungcan.2012.10.007 [published Online First: 2012/11/21]
150. Hotta K, Kato Y, Leighl N, et al. Magnitude of the benefit of progression-free survival as a potential surrogate marker in phase 3 trials assessing targeted agents in molecularly selected patients with advanced non-small cell lung cancer: systematic review. *PLoS One* 2015;10(3):e0121211. doi: 10.1371/journal.pone.0121211 [published Online First: 2015/03/17]

©2022 Zhang Y et al. *JAMA Network Open*.

151. Aboshi M, Kaneko M, Narukawa M. Factors affecting the association between overall survival and progression-free survival in clinical trials of first-line treatment for patients with advanced non-small cell lung cancer. *J Cancer Res Clin Oncol* 2014;140(5):839-48. doi: 10.1007/s00432-014-1617-3 [published Online First: 2014/02/25]
152. Suzuki H, Hirashima T, Okamoto N, et al. Relationship between progression-free survival and overall survival in patients with advanced non-small cell lung cancer treated with anticancer agents after first-line treatment failure. *Asia Pac J Clin Oncol* 2015;11(2):121-8. doi: 10.1111/ajco.12199 [published Online First: 2014/05/09]
153. Ng R, Pond GR, Tang PA, et al. Correlation of changes between 2-year disease-free survival and 5-year overall survival in adjuvant breast cancer trials from 1966 to 2006. *Ann Oncol* 2008;19(3):481-6. doi: 10.1093/annonc/mdm486 [published Online First: 2007/11/22]
154. Berruti A, Amoroso V, Gallo F, et al. Pathologic complete response as a potential surrogate for the clinical outcome in patients with breast cancer after neoadjuvant therapy: a meta-regression of 29 randomized prospective studies. *J Clin Oncol* 2014;32(34):3883-91. doi: 10.1200/jco.2014.55.2836 [published Online First: 2014/10/29]
155. Cortazar P, Zhang L, Untch M, et al. Pathological complete response and long-term clinical benefit in breast cancer: the CTNeoBC pooled analysis. *Lancet* 2014;384(9938):164-72. doi: 10.1016/s0140-6736(13)62422-8 [published Online First: 2014/02/18]
156. Petrelli F, Barni S. Surrogate endpoints in metastatic breast cancer treated with targeted therapies: an analysis of the first-line phase III trials. *Med Oncol* 2014;31(1):776. doi: 10.1007/s12032-013-0776-4 [published Online First: 2013/11/26]
157. Adunlin G, Cyrus JW, Dranitsaris G. Correlation between progression-free survival and overall survival in metastatic breast cancer patients receiving anthracyclines, taxanes, or targeted therapies: a trial-level meta-analysis. *Breast Cancer Res Treat* 2015;154(3):591-608. doi: 10.1007/s10549-015-3643-5 [published Online First: 2015/11/26]
158. Wilkerson J, Fojo T. Progression-free survival is simply a measure of a drug's effect while administered and is not a surrogate for overall survival. *Cancer J* 2009;15(5):379-85. doi: 10.1097/PPO.0b013e3181bef8cd [published Online First: 2009/10/15]
159. Sherrill B, Amonkar M, Wu Y, et al. Relationship between effects on time-to-disease progression and overall survival in studies of metastatic breast cancer. *Br J Cancer* 2008;99(10):1572-8. doi: 10.1038/sj.bjc.6604759 [published Online First: 2008/11/13]
160. Beauchemin C, Cooper D, Lapierre M, et al. Progression-free survival as a potential surrogate for overall survival in metastatic breast cancer. *Onco Targets Ther* 2014;7:1101-10. doi: 10.2147/ott.S63302 [published Online First: 2014/06/28]
161. Liu L, Chen F, Zhao J, et al. Correlation between overall survival and other endpoints in metastatic breast cancer with second- or third-line chemotherapy: Literature-based analysis of 24 randomized trials. *Bull Cancer* 2016;103(4):336-44. doi: 10.1016/j.bulcan.2016.01.002 [published Online First: 2016/02/15]
162. Petrelli F, Barni S. Surrogate end points and postprogression survival in renal cell carcinoma: an analysis of first-line trials with targeted therapies. *Clin Genitourin Cancer* 2013;11(4):385-9. doi: 10.1016/j.clgc.2013.07.012 [published Online First: 2013/10/08]
©2022 Zhang Y et al. *JAMA Network Open*.

163. Bria E, Massari F, Maines F, et al. Progression-free survival as primary endpoint in randomized clinical trials of targeted agents for advanced renal cell carcinoma. Correlation with overall survival, benchmarking and power analysis. *Crit Rev Oncol Hematol* 2015;93(1):50-9. doi: 10.1016/j.critrevonc.2014.08.001 [published Online First: 2014/09/10]
164. Delea TE, Khuu A, Heng DY, et al. Association between treatment effects on disease progression end points and overall survival in clinical studies of patients with metastatic renal cell carcinoma. *Br J Cancer* 2012;107(7):1059-68. doi: 10.1038/bjc.2012.367 [published Online First: 2012/09/01]
165. Johnson KR, Liauw W, Lassere MN. Evaluating surrogacy metrics and investigating approval decisions of progression-free survival (PFS) in metastatic renal cell cancer: a systematic review. *Ann Oncol* 2015;26(3):485-96. doi: 10.1093/annonc/mdu267 [published Online First: 2014/07/25]
166. Ciani O, Buyse M, Garside R, et al. Meta-analyses of randomized controlled trials show suboptimal validity of surrogate outcomes for overall survival in advanced colorectal cancer. *J Clin Epidemiol* 2015;68(7):833-42. doi: 10.1016/j.jclinepi.2015.02.016 [published Online First: 2015/04/13]
167. Sidhu R, Rong A, Dahlberg S. Evaluation of progression-free survival as a surrogate endpoint for survival in chemotherapy and targeted agent metastatic colorectal cancer trials. *Clin Cancer Res* 2013;19(5):969-76. doi: 10.1158/1078-0432.Ccr-12-2502 [published Online First: 2013/01/11]
168. Chirila C, Odom D, Devercelli G, et al. Meta-analysis of the association between progression-free survival and overall survival in metastatic colorectal cancer. *Int J Colorectal Dis* 2012;27(5):623-34. doi: 10.1007/s00384-011-1349-7 [published Online First: 2011/11/15]
169. Giessen C, Laubender RP, Ankerst DP, et al. Surrogate endpoints in second-line treatment for mCRC: a systematic literature-based analysis from 23 randomised trials. *Acta Oncol* 2015;54(2):187-93. doi: 10.3109/0284186x.2014.938830 [published Online First: 2014/07/16]
170. Cremolini C, Antoniotti C, Pietrantonio F, et al. Surrogate Endpoints in Second-Line Trials of Targeted Agents in Metastatic Colorectal Cancer: A Literature-Based Systematic Review and Meta-Analysis. *Cancer Res Treat* 2017;49(3):834-45. doi: 10.4143/crt.2016.249 [published Online First: 2016/11/20]
171. Siddiqui MK, Tyczynski J, Pahwa A, et al. Objective response rate is a possible surrogate endpoint for survival in patients with advanced, recurrent ovarian cancer. *Gynecol Oncol* 2017;146(1):44-51. doi: 10.1016/j.ygyno.2017.03.515 [published Online First: 2017/04/12]
172. Abdel-Rahman O. Surrogate end points for overall survival in trials of PD-(L)1 inhibitors for urinary cancers: a systematic review. *Immunotherapy* 2018;10(2):139-48. doi: 10.2217/imt-2017-0115 [published Online First: 2017/12/21]
173. Xie W, Regan MM, Buyse M, et al. Metastasis-Free Survival Is a Strong Surrogate of Overall Survival in Localized Prostate Cancer. *J Clin Oncol* 2017;35(27):3097-104. doi: 10.1200/jco.2017.73.9987 [published Online First: 2017/08/11]
174. Smith MR, Mehra M, Nair S, et al. Relationship Between Metastasis-free Survival and Overall Survival in Patients With Nonmetastatic Castration-resistant Prostate Cancer. *Clin Genitourin Cancer* 2020;18(2):e180-e89. doi: 10.1016/j.clgc.2019.10.030 [published Online First: 2020/01/26]
175. Sheth M, Ko J. Exploring the relationship between Overall Survival (OS), Progression Free Survival (PFS) and Objective Response Rate (ORR) in patients with advanced melanoma. *Cancer Treat Res Commun* 2021;26:100272. doi: 10.1016/j.ctarc.2020.100272 [published Online First: 2020/12/19]

©2022 Zhang Y et al. *JAMA Network Open*.

176. Nie RC, Yuan SQ, Wang Y, et al. Surrogate endpoints for overall survival in anti-programmed death-1 and anti-programmed death ligand 1 trials of advanced melanoma. *Ther Adv Med Oncol* 2020;12:1758835920929583. doi: 10.1177/1758835920929583 [published Online First: 2020/07/01]
177. Penel N, Cousin S, Duhamel A, et al. Activity endpoints reported in soft tissue sarcoma phase II trials: quality of reported endpoints and correlation with overall survival. *Crit Rev Oncol Hematol* 2013;88(2):309-17. doi: 10.1016/j.critrevonc.2013.05.004 [published Online First: 2013/05/28]
178. Tykocki T, Eltayeb M. Ten-year survival in glioblastoma. A systematic review. *J Clin Neurosci* 2018;54:7-13. doi: 10.1016/j.jocn.2018.05.002 [published Online First: 2018/05/29]
179. Ballman KV, Buckner JC, Brown PD, et al. The relationship between six-month progression-free survival and 12-month overall survival end points for phase II trials in patients with glioblastoma multiforme. *Neuro Oncol* 2007;9(1):29-38. doi: 10.1215/15228517-2006-025 [published Online First: 2006/11/17]
180. Llovet JM, Montal R, Villanueva A. Randomized trials and endpoints in advanced HCC: Role of PFS as a surrogate of survival. *J Hepatol* 2019;70(6):1262-77. doi: 10.1016/j.jhep.2019.01.028 [published Online First: 2019/04/04]
181. Xu H, Cao D, Zheng Y, et al. Potential predictors for survival in hepatocellular carcinoma patients treated with immune checkpoint inhibitors: A meta-analysis. *Int Immunopharmacol* 2021;100:108135. doi: 10.1016/j.intimp.2021.108135 [published Online First: 2021/09/17]

©2022 Zhang Y et al. *JAMA Network Open*.
